# Dendritic cell-derived IL-27 p28 regulates T cell program in pathogenicity and alleviates acute graft-versus-host disease

**DOI:** 10.1038/s41392-022-01147-z

**Published:** 2022-09-16

**Authors:** Huanle Gong, Shoubao Ma, Jia Chen, Bingyu Yang, Shuangzhu Liu, Xin Liu, Jingjing Han, Xiaojin Wu, Lei Lei, Zhinan Yin, Hongjian Sun, Di Yu, Haiyan Liu, Yang Xu, Depei Wu

**Affiliations:** 1 The First Affiliated Hospital of Soochow University, National Clinical Research Center for Hematologic Diseases, Jiangsu Institute of Hematology, Institute of Blood and Marrow Transplantation, Soochow University, Suzhou, 215123 China; 2 Collaborative Innovation Center of Hematology, Soochow University, Suzhou, 215123 China; 3 Department of Hematology, The First Affiliated Hospital of Xi’an Jiaotong University, Xi’an, Shaanxi 710061 China; 4 Zhuhai Precision Medical Center, Zhuhai People’s Hospital (Zhuhai Hospital Affiliated with Jinan University), Jinan University, Zhuhai, 519000 Guangdong China; 5 The Biomedical Translational Research Institute, Faculty of Medical Science, Jinan University, Guangzhou, 510632 Guangdong China; 6 Shandong Artificial Intelligence Institute, Qilu University of Technology (Shandong Academy of Sciences), Jinan, 250200 China; 7 The University of Queensland Diamantina Institute, Faculty of Medicine, The University of Queensland, Brisbane, QLD 4072 Australia; 8 Immunology Programme, Life Sciences Institute; Immunology Translational Research Program and Department of Microbiology and Immunology, Yong Loo Lin School of Medicine, National University of Singapore, Singapore, 117456 Singapore

**Keywords:** Bone marrow transplantation, Transplant immunology, Adaptive immunity

## Abstract

Interleukin 27 (IL-27), a heterodimeric cytokine composed of Epstein-Barr virus-induced 3 and p28, is a pleiotropic cytokine with both pro-and anti-inflammatory properties. However, the precise role of IL-27 in acute graft-*versus*-host disease is not yet fully understood. In this study, utilizing mice with IL-27 p28 deficiency in dendritic cells (DCs), we demonstrated that IL-27 p28 deficiency resulted in impaired Treg cell function and enhanced effector T cell responses, corresponding to aggravated aGVHD in mice. In addition, using single-cell RNA sequencing, we found that loss of IL-27 p28 impaired Treg cell generation and promoted IL-1R2^+^TIGIT^+^ pathogenic CD4^+^ T cells in the thymus at a steady state. Mechanistically, IL-27 p28 deficiency promoted STAT1 phosphorylation and Th1 cell responses, leading to the inhibition of Treg cell differentiation and function. Finally, patients with high levels of IL-27 p28 in serum showed a substantially decreased occurrence of grade II-IV aGVHD and more favorable overall survival than those with low levels of IL-27 p28. Thus, our results suggest a protective role of DC-derived IL-27 p28 in the pathogenesis of aGVHD through modulation of the Treg/Teff cell balance during thymic development. IL-27 p28 may be a valuable marker for predicting aGVHD development after transplantation in humans.

## Introduction

Acute graft-*versus*-host disease (aGVHD), one of the major complications early after allogeneic hematopoietic stem cell transplantation (allo-HSCT), is characterized by host tissue injury mediated mainly by donor T cells following interaction with either donor- or host-derived antigen-presenting cells (APCs).^1,2^ Preconditioning induces tissue damage and leads to the secretion of massive pro-inflammatory cytokines by the donor or host APCs, including IL-1β, IL-6, IL-12, and TNF-α, which then trigger the activation and proliferation of alloreactive T cells and subsequently promote donor T cell polarization toward Th1, Th2, or Th17 phenotypes.^3^ These cells further contribute to the pathogenesis of aGVHD by their effector cytokines, such as IFN-γ, IL-4, or IL-17A.^4^ Of note, several therapeutic approaches targeting cytokines have already shown promising outcomes for aGVHD prevention.^5,6^ Therefore, a better understanding of the pathophysiology of aGVHD could help to develop novel strategies for the prevention and treatment of aGVHD.

IL-27, a heterodimeric cytokine composed of EBI3 and p28, signals through binding with IL-27 receptor formed by IL-27Rα (also named as WSX1) and gp130.^7^ It has been reported that p28 subunit could be secreted independently of EBI3 and conversely antagonize the IL-27 signaling.^8^ IL-27 is predominantly produced by activated APCs, including DCs, monocytes, and macrophages.^7^ IL-27Rα is constitutively expressed among a range of cell types including T cells, B cells, intestinal epithelial cells as well as hematopoietic stem cells.^9,10^ Engagement of IL-27 and IL-27 receptor transduces cellular signals via JAK1/2 and STAT1/3 pathways.^11^ IL-27 was recognized as an inflammatory cytokine with potent immune regulatory properties. On the one hand, IL-27 was early characterized by the promotion of IFN-γ production by NK cells and naive CD4^+^ T cells.^12^ IL-27 also induced T-bet expression to facilitate Th1 cell function.^13^ Likewise, IL-27Rα was essential for Th1 cell differentiation in infectious diseases.^14^ Endogenous IL-27 p28 produced by DCs and monocytes increased antigen-specific CD8^+^ T cells expansion and their effector function in vaccine-elicited cellular immunity.^15^ On the other hand, IL-27 exhibited immune inhibitory roles by promoting IL-10 release in Treg, T regulatory type 1 (Tr1), IFN-γ^+^T-bet^+^Foxp3^−^ Th1, Th2, and Th17 cells.^8,16–18^ T cells deficient in IL-27Rα failed to produce IL-10 by TCR stimulation.^19^ Mice with IL-27Rα ablation had a high susceptibility to experimental autoimmune encephalomyelitis (EAE), which was associated with enhanced IL-17A production.^20^ Concordantly, Treg cell-specific depletion of IL-27Rα argumented EAE development by impairing Treg cell stability.^21^ Moreover, IL-27 upregulated the expression of a diversity of inhibitory molecules on T cells, including programmed death ligand 1 (PD-L1), lymphocyte activation gene-3 (Lag3), T cell immunoglobulin domain and mucin domain 3 (Tim3) and stem cell antigen-1 (Sca-1).^8^ IL-27 produced by innate-like natural regulatory B1-a cells (i27-Breg) prevents neuroinflammation through upregulating PD-1 and Lag3 and thereby suppressing Th1/17 responses.^22^ A subset of antigen-specific Foxp3^−^CD4^+^T cells could also produce IL-27 upon malaria parasite infections.^23^ A very recent study showed that CX3CR1^+^ cells produced IL-27 and restrained obesity in mice.^24^ However, the exact cellular source of IL-27 during the process of aGVHD development has not been fully addressed. Recent studies indicated that IL-27 p28 deficiency aggravated, whereas blockage of IL-27 p28 signaling alleviated aGVHD in murine models.^25,26^ However, a lack of IL-27Rα expression on donor T cells reduced Th1 responses and mitigated aGVHD.^25,27^ Therefore, the role of IL-27 signaling in aGVHD remains controversial. Moreover, the expression pattern and the clinical significance of IL-27 in aGVHD patients remain unknown.

Treg cells are central regulators of immune response and tolerance, which potently suppress both acute and chronic GVHD.^28,29^ It has been well accepted that IL-27 regulates Treg cell differentiation and functions in malignancy, allergic pathologies and autoimmune disorders. IL-27 gene therapy-induced melanoma rejection by inhibiting Treg cell function in mice .^30^ Accordingly, IL-27 restrained Treg cell generation and promoted colitis induced by CD4^+^CD45Rb^hi^ T cells.^31^ However, IL-27 promoted Lag3 expression on Tregs and attenuated antigen-induced allergic inflammatory response.^32^ IL-27 also drived T-bet^+^CXCR3^+^ Treg cell expansion and limited Th1 cell type infections.^33^ IL-27 or IL-27Rα deficient mice have no changes in Treg development, suggesting IL-27 does not affect Treg homeostasis at a steady state.^20^ As in the context of aGVHD, IL-27 pre-stimulation enforced iTreg function, while IL-27Rα expression inhibited Treg development after allo-BMT.^27,34^ Thus, the complicated role of IL-27 signal in Treg regulation upon aGVHD remains to be investigated in detail.

In this study, we found that IL-27 p28 was predominantly produced by DCs in aGVHD and DC-specific deficiency of IL-27 p28 mitigated aGVHD by regulating T cell program in pathogenicity in a cell-intrinsic manner. IL-27 p28 deficiency inhibited Th1 responses and promoted the differentiation and inhibitory ability of Treg cells via the IFN-γ/STAT1 signaling pathway. IL-27 p28 deficiency impaired Treg generation and promoted IL-1R2^+^TIGIT^+^ pathogenic CD4^+^ T cells in the thymus at a steady state. We also observed that high serum levels of IL-27 p28 were associated with a lower incidence of severe aGVHD in patients who underwent allo-HSCT, suggesting that DC-derived IL-27 p28 protects against aGVHD development and might be a potential prognostic marker for severe aGVHD after allo-HSCT.

## Results

### IL-27 p28 deficiency aggravates aGVHD in mice

**Fig. 1:**
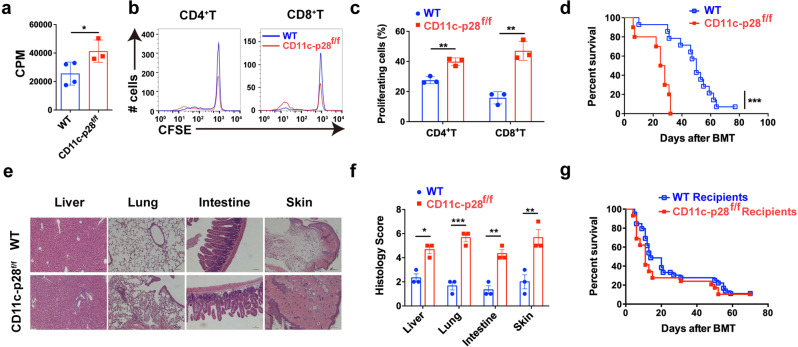
IL-27 p28 deficiency aggravates aGVHD in mice. **a**–**c** BALB/c DCs were cocultured with CFSE-labeled T cells (ratio 1:10) from CD11c-p28^f/f^ mice or control littermates, respectively. Proliferations were assessed by **a** ^3^H-TdR or **b**, **c** flow cytometry 5 days post coculture. **d**–**f** BALB/c recipients were transplanted with 1 × 10^7^ WT BMs and 5 × 10^6^ splenocytes from either WT or CD11c-p28^f/f^ mice (*n* = 10–14 per group). Overall survival curve is depicted (**d**). Representative H&E stained sections and histological scores of aGVHD tissues from recipients 14 days post-transplantation are shown (**e**, **f**). **g** C57BL/6 or CD11c-p28^f/f^ recipients were lethal irradiation and received 1 × 10^7^ BMs and 7.5 × 10^7^ splenocytes from BALB/c mice (*n* = 15 per group). The overall survival curve is depicted. Data are representative of three independent experiments and presented as mean ± SD. *P < 0.05; **P < 0.01; ***P < 0.001

To address the physiological role of IL-27 p28 in aGVHD pathogenesis, we first assessed the cellular source of IL-27 p28 during aGVHD. Results showed that DCs, rather than T cells, neutrophils, or monocytes, were the predominant source of IL-27 p28 in both murine aGVHD models and patients at the onset of aGVHD (Supplementary Fig. 1a, b). We therefore generated IL-27 p28 conditional knockout (p28 cKO) mice in DCs by crossing p28^f/f^ mice with CD11c Cre mice.^35^ IL-27 p28 expression in the serum was markedly reduced in CD11c-p28^f/f^ mice compared with WT mice at a steady-state or on day 7 after allogenic-bone marrow transplantation (BMT) (Supplementary Fig. 1c, d). Moreover, the levels of IL-27 heterodimer were also reduced in CD11c-p28^f/f^ mice compared with WT mice (Supplementary Fig. 1e, f). In vitro mixed lymphocyte reaction (MLR) revealed that T cells from p28 cKO mice displayed aggravated alloreactivity than those from WT mice (Fig. 1a), as evidenced by enhanced cell proliferation (Fig. 1b, c). We then established an MHC-mismatched murine aGVHD model using CD11c-p28^f/f^ or WT mice as donors. Compared with WT recipients, mice that received CD11c-p28^f/f^ splenocytes showed accelerated aGVHD mortality (Fig. 1d). Histological analysis also revealed that recipients of CD11c-p28^f/f^ splenocytes showed much more severe tissue damage (Fig. 1e, f). However, the mortality showed no significant difference when CD11c-p28^f/f^ mice were used as recipients (Fig. 1g). Collectively, these results demonstrate that donor-derived IL-27 p28 protects from aGVHD development in mice.

### Loss of IL-27 p28 enhances T cell responses after allo-HSCT

**Fig. 2:**
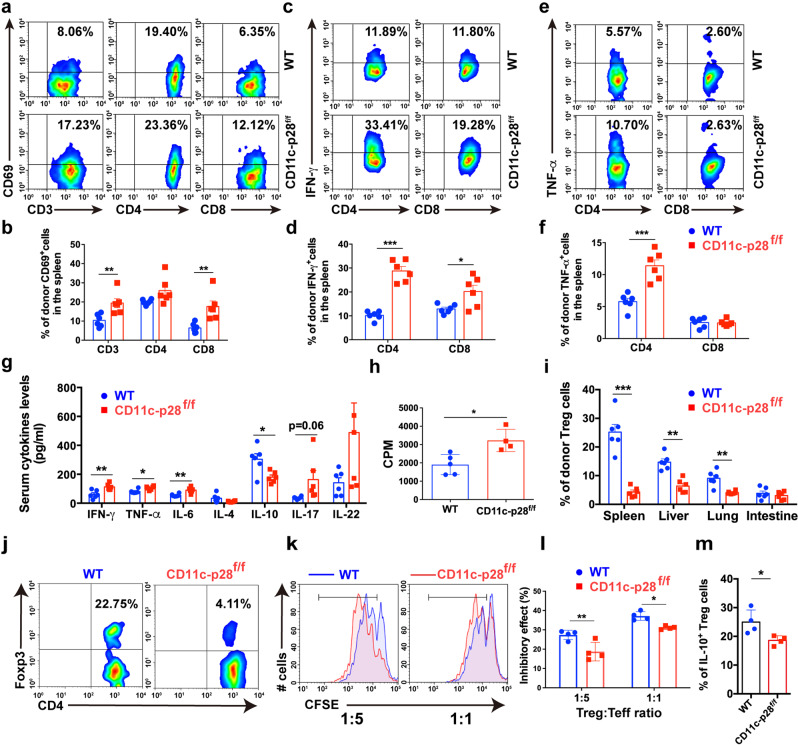
Loss of IL-27 p28 enhances T cell responses after allo-HSCT. **a**, **b** BALB/c recipients were transplanted with 1 × 10^7^ WT BMs together with 5 × 10^6^ splenocytes from either C57BL/6 or CD11c-p28^f/f^ mice. Immune cell subsets were examined 14 days post-transplantation. Representative flow cytometric plots and quantification of activated T cells in spleens (among H2-Kb^+^H2-Kd^-^ cells) from recipients (*n* = 6 per group) are depicted. **c**–**f** Representative flow cytometric plots and quantification of IFN-γ-producing T cells (**c**, **d**) and TNF-α-producing T cells (**e**, **f**) in spleens (among H2-Kb^+^H2-Kd^-^ cells) from recipients (*n* = 6 per group) are depicted. **g** Serum from BALB/c recipients was collected 14 days post-transplantation and cytokines production was examined using LEGENDplex (*n* = 5–6 per group). **h** Splenocytes from aGVHD recipients were cocultured with irradiated BALB/c splenocytes. Proliferation rate was detected by ^3^H-TdR incorporation assay 3 days after coculture. **i**, **j** Treg cells were detected 14 days post-transplantation via FACS. Quantitative data (**i**) and representative plots (**j**) of donor Tregs are shown (*n* = 6 per group). **k**, **l** CFSE-labeled effector T cells (CD4^+^CD25^−^ T cells) were cocultured with Treg cells sorted from the spleens of C57BL/6 or CD11c-p28^f/f^ mice at the indicated ratios for 5 days. Representative figures (**k**) and frequency of cell proliferation are depicted (**l**) (*n* = 4 per group). **m** The frequency of IL-10^+^ Treg cells is shown (*n* = 4 per group). Data are representative of three independent experiments and presented as mean ± SD. *P < 0.05; **P < 0.01; ***P < 0.001

To investigate the mechanisms by which IL-27 p28 alleviates aGVHD, we profiled the immune cell responses in aGVHD target tissues post transplantation. Donor T cells were substantially elevated in aGVHD target tissues from recipients that received CD11c-p28^f/f^ grafts compared with those received WT grafts (Supplementary Fig. 2a). Notably, T cells from recipients transplanted with CD11c-p28^f/f^ splenocytes showed more activated phenotypes, indicated by increased CD69 expression compared with those from WT recipients (Fig. 2a, b and Supplementary Fig. 2b). Furthermore, the frequencies of IFN-γ- and TNF-α-production in donor T cells were significantly upregulated in recipients of CD11c-p28^f/f^ grafts compared with WT controls (Fig. 2c–f and Supplementary Fig. 2c). However, the IL-17A and IL-4 expression had no changes in aGVHD target tissues (Supplementary Fig. 2d). In addition, productions of IL-6, IFN-γ, and TNF-α were significantly upregulated, whereas IL-10 was downregulated in recipients of CD11c-p28^f/f^ grafts compared with those that received WT grafts (Fig. 2g). Furthermore, recipients of CD11c-p28^f/f^ grafts exhibited enhanced donor T cell alloreactivity in vivo (Fig. 2h). These findings demonstrate that loss of IL-27 p28 leads to enhanced alloreactive T cell responses which accelerates the aGVHD-related mortality.

Tregs suppress conventional T cell activation and control GVHD.^36^ In our models, we observed that Tregs were substantially decreased in aGVHD target tissues of recipients that received CD11c-p28^f/f^ grafts compared with those received WT grafts (Fig. 2i, j). However, CD11c-p28^f/f^ Treg cells exhibited a similar apoptosis rate to that of WT recipients (Supplementary Fig. 2e), indicating that IL-27 p28 deficiency did not affect Treg cell survival in vivo. In contrast, Treg cells isolated from recipients of CD11c-p28^f/f^ grafts displayed impaired suppressive effects toward CD4^+^ Teff cells compared to those from WT recipients (Fig. 2k, l). IL-27 has been identified as a differentiation factor that contributes to Tr1 cell generation.^16,19,37^ We found that IL-10 levels were significantly downregulated in the serum of recipients of CD11c-p28^f/f^ grafts (Fig. 2g). However, no significant changes of Tr1 populations were observed in aGVHD target tissues between these two groups (Supplementary Fig. 2f), suggesting that the reduced IL-10 levels may be due to the decreased Treg cells. Indeed, IL-10 producing-Treg cells were significantly reduced in recipients of CD11c-p28^f/f^ grafts (Fig. 2m). Together, these results demonstrate that the exacerbation of aGVHD mediated by IL-27 p28 deficiency associates with defective Treg cell function, which fails to suppress the alloreactive T cell responses.

### Donor DC-derived IL-27 p28 deficiency and intrinsic functional defects of T cells are both responsible for exacerbated aGVHD

**Fig. 3:**
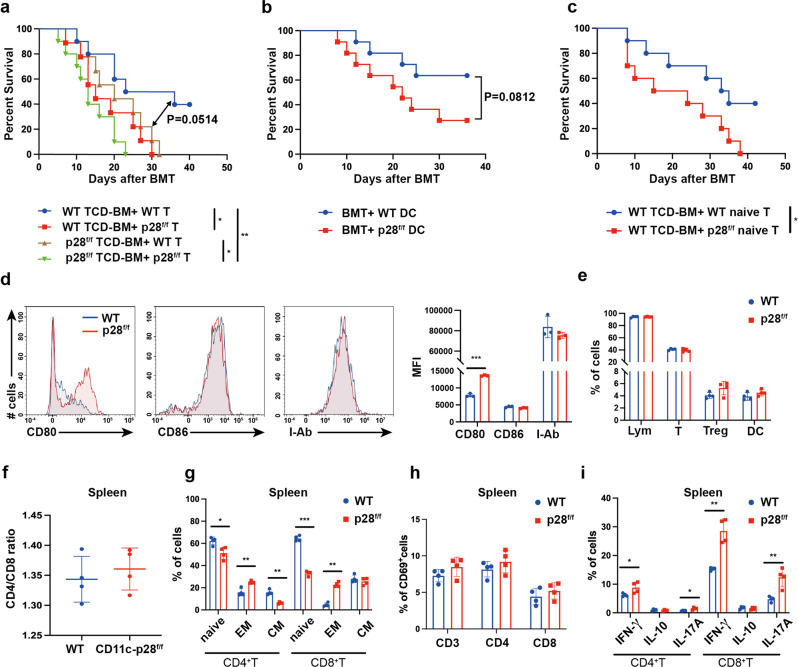
Donor DC-derived IL-27 p28 deficiency and intrinsic functional defects of T cells are both responsible for exacerbated aGVHD. **a** BALB/c recipients received either WT or CD11c-p28^f/f^ allografts of 5 × 10^6^ TCD-BMs and 1 × 10^6^ T cells as indicated (*n* = 10 per group). **b** WW recipients were injected with either 1 × 10^6^ WT DCs or CD11c-p28^f/f^ DCs at day 0, day1, and day2 post-BMT (*n* = 10 per group). The overall survival curve is depicted. **c** BALB/c recipients received 5 × 10^6^ TCD-BMs from WT mice together with 1 × 10^6^ naive T cells from WT or CD11c-p28^f/f^ mice, respectively (*n* = 10 per group). **d**–**i** Splenocytes from normal donors were detected by flow cytometry. **d** CD80, CD86, and MHC-II expression on DCs. **e** Percentages of lymphocytes, CD3^+^ T, Tregs, and DCs. **f** The ratio of T cells in the spleen. **g** Percentages of T cell suesets. **h** CD69 expression on T cells. **i** IFN-γ, IL-17A, and IL-10 expression in T cells (*n* = 4 per group). Data are representative of three independent experiments and presented as mean ± SD. *P < 0.05; **P < 0.01; ***P < 0.001

Based on our results, three hypotheses may explain the enhanced aGVHD by IL-27 p28 deficiency in CD11c positive cells. First, donor CD11c^+^ DC-derived IL-27 p28 directly affects allogenic-T cell activity during aGVHD. Second, splenic T cells from p28 cKO mice may have unleashed effector functions before transferring to aGVHD receipt. Third, a recent study reported that a certain subset of T cells also express CD11c.^23^ This minor population of T cells acquired IL-27 p28 deficiency may affect aGVHD development. To clarify whether donor T cells or DCs contribute to the effect of IL-27 p28, we transplanted recipients with T cell-depleted (TCD)-BMs and purified T cells from either WT or CD11c-p28^f/f^ mice. As shown in Fig. 3a, recipients received TCD-BM from WT mice together with T cells from CD11c-p28^f/f^ mice (hereinafter referred to as WK) had significantly shortened survival compared with those received TCD-BM and T cells both from WT mice (hereinafter referred to as WW). These results were consistent with our model in which BM and splenocytes were used as grafts, suggesting that T cells from p28 cKO mice are more pathogenic than those from WT mice, which contribute to the exacerbated aGVHD. Recipients of TCD-BM from CD11c-p28^f/f^ mice and T cells from WT mice (hereinafter referred to as KW) showed a trend of shortened survival compared with the WW group (*P* = 0.0514), suggesting that donor DC-derived IL-27 p28 deficiency also contributes to the exacerbated aGVHD, albeit to a lesser extent (Fig. 3a). To mechanistically interrogate the role of DC-derived IL-27 p28 in aGVHD, we co-transferred bone marrow-derived DCs (BMDCs) from either WT or CD11c-p28^f/f^ mice into WW recipients. We observed that co-transfer of BMDCs from CD11c-p28^f/f^ mice exacerbated aGVHD, compared with those from WT mice, although the difference was not statistically significant (*P* = 0.082, Fig. 3b). These results indicate that donor DC-derived IL-27 p28 indeed contributes to the aGVHD development. To further clarify whether the aggravated aGVHD are due to pre-activation and distribution of T cell cargo before transfer or an altered differentiation upon transplantation, we compared the effects of donor-derived naive T cells in aGVHD induction. Interestingly, naive T cells from CD11c-p28^f/f^ mice significantly enhanced aGVHD development than those from WT mice (Fig. 3c), indicating that the intrinsic functional defects occur before T cells are activated. We also observed an upregulation of co-stimulatory molecule CD80 expression on DCs (Fig. 3d). In addition, recipients transplanted with TCD-BM and T cells both from CD11c-p28^f/f^ mice had the shortest survival compared with WW (*P* = 0.004) and KW groups (*P* = 0.04), and showed a trend of shorter survival than WK group (*P* = 0.1698). These results indicate that DC-derived IL-27 p28 and T cells of p28 cKO donors are both responsible for aGVHD pathogenesis.

We found that T cells at a steady-state did not express CD11c (<0.5%) but had very low levels in vitro (about 2% among total T cells) with allo-stimulation or in vivo (about 1% among total T cells) at 7 days after allo-BMT in both WT and CD11c-p28^f/f^ mice (Supplementary Fig. 3a-c). Both CD11c^+^ and CD11c^−^ T cells did not secrete any detectable IL-27 p28 (data not shown). In addition, the IFN-γ production by CD11c^+^ and CD11c^−^ T cells were comparable between WT and CD11c-p28^f/f^ mice under allo-stimulation (Supplementary Fig. 3d, e). Thus, CD11c^+^ T cell-deficient of IL-27 p28 unlikely contributes to aGVHD development. Of note, although WT and p28 cKO mice exhibited the equivalent levels of T cells, Treg cells, DCs, and CD4/CD8 ratio in the spleen at a steady-state (Fig. 3e, f), T cells from p28 cKO donors showed a higher activation phenotype, as evidenced by increased effector memory T cell populations (Fig. 3g). However, CD69 expression showed no difference among these T cells (Fig. 3h). Furthermore, increased proportions of IL-17A and IFN-γ producing T cells were observed in p28 cKO mice (Fig. 3i), indicating that T cells from p28 cKO mice are intrinsically different from those T cells from WT mice. DC-derived IL-27 p28 may mediate aGVHD development by affecting T cell pathogenicity during allo-HSCT.

### IL-27 p28 deficiency impairs Treg cell generation and promotes pathogenic IL-1R2^+^TIGIT^+^ CD4^+^T cells in the thymus

**Fig. 4:**
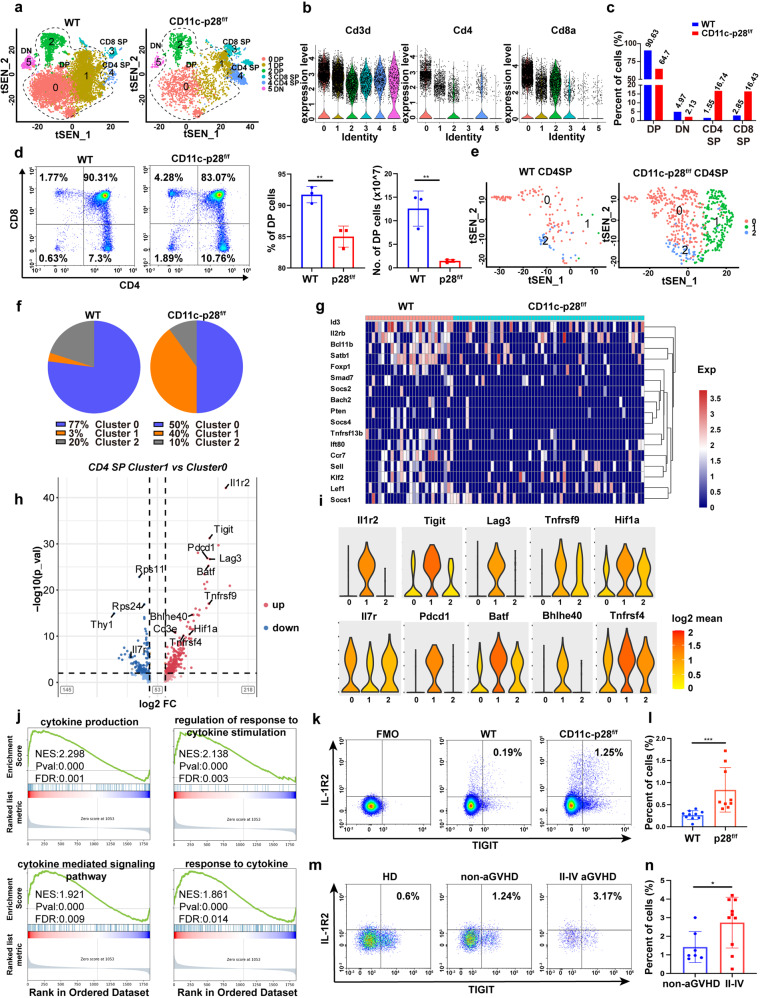
IL-27 p28 deficiency impairs Treg cell generation and promotes pathogenic IL-1R2^**+**^TIGIT^**+**^ CD4^**+**^T cells in the thymus. **a** Reclustering of T cell subpopulations in WT and CD11c-p28^f/f^ mice. **b** Violin plots of the relative expressions of CD3D, CD4, and CD8. **c** Percent of different thymic T cell subsets determined by scRNA-seq analysis. **d** Populations and numbers of DP T cells in the thymus from WT and CD11c-p28^f/f^ mice by flow cytometry. **e** t-SNE visualization of CD4 SP T cells. **f** Components of subclusters in CD4 SP cells. **g** Expression profile of genes involved in Treg development. **h** Volcano plot showing differential gene expression between Cluster 1 and Cluster 0 cells from CD11c-p28^f/f^ and WT mice. **i** Violin plots showing the expression profile of T cell effector genes. Expression is measured as the log2-fold change. **j** GSEA of the upregulated gene set in Cluster 1 versus Cluster 0 in CD4 SP cells from CD11c-p28^f/f^ relative to WT mice. **k**, **l** Populations of donor-derived IL-1R2^+^TIGIT^+^CD4^+^T cells were detected by FACS 7 days post-transplantation (*n* = 9–10 per group). **m**, **n** Percentages of IL-1R2^+^TIGIT^+^CD4^+^T cells in PBMCs were detected by FACS 30 days post-allo-HSCT. *P < 0.05; **P < 0.01; ***P < 0.001

Abnormalities in thymic development at least partially limites T cell maturation and functions.^38^ Our results are in agreement with prior studies in which DC-derived IL-27 p28 regulated T cell development in the thymus.^35,39^ However, the mechanism by which IL-27 p28 regulates thymic T cell differentiation remains undetermined. To address this, we applied single-cell RNA sequencing (scRNA-seq) to thymocytes collected from WT and CD11c-p28^f/f^ mice, which allowed highly detailed profiling of immune cell subsets during development at the single-cell level. After quality control and filtering out poor-quality cells, a whole-transcriptome database of 17922 cells from the two groups was analyzed. We identified 9 clusters by t-Distributed Stochastic Neighbor Embedding (t-SNE), including T cells, B cells, DCs, macrophages, and fibroblasts (Supplementary Fig. 4a-c). T cells with specific expression of *CD3d*, *CD4*, or *CD8a* were distinguished from other clusters and were reclustered for further analysis (Fig. 4a). Violin plots revealed that cluster 5 represented double-negative (DN) T cells; clusters 0, 1, and 2 represented double-positive (DP) T cells; and clusters 3 and 4 represented CD8 and CD4 single-positive (SP) T cells, respectively (Fig. 4b). We observed a reduction in DP cells and elevation in CD4 and CD8 SP cells in IL-27 p28-deficient mice compared to WT mice (Fig. 4c). Flow cytometry confirmed that CD11c-p28^f/f^ mice had reduced DP and increased CD4 and CD8 SP T cells compared with WT mice (Fig. 4d). We further divided CD4 SP T cells into three subclusters (Fig. 4e). Principal component analysis (PCA) showed a difference among these three clusters (Supplementary Fig. 5a). Further analysis showed that cluster 0 and cluster 1 represented conventional T cells, which was indicated by low expressions of *Foxp3*. Cluster 2 represented Treg cells, which was designated by high levels of *Foxp3* and *IL2ra* expression (Supplementary Fig. 5b). CD4_cluster 0 and 2 were decreased dramatically in IL-27 p28-deficient mice (Fig. 4f), indicating that IL-27 p28 deficiency impairs Treg cell generation during thymic development. The expression levels of genes involved in Treg cell development, including *Sell*, *Ccr7*, *Tnfrsf13b*, *Bcl11b*, *Stab1*, *Id3*, *Lef1*, and *Bach2* were pronouncedly reduced in Tregs from CD11c-p28^f/f^ mice compared to those from WT mice (Fig. 4g).^40–47^ Subsequently, we found that the percentage of CD4_cluster 1 was 3% in WT mice while markedly increased to 40% in CD11c-p28^f/f^ mice. Surprisingly, this T cell cluster was hyperactivated, with highly expressed activation marker genes, such as *Tnfrsf9*, *Tight*, *Lag3*, *Pdcd1*, *Batf*, *Maf*, *Bhlhe40*, *Tnfrsf18*, and *Tnfrsf4*, and downregulation of the naive gene *Il7r*.^48^ In addition, *Il1r2* and *Tigit* were highly and specifically expressed in Cluster 1 (Fig. 4h, i). We therefore named cluster 1 cells as IL-1R2^+^TIGIT^+^ activated CD4^+^T cells. Flow cytometry data confirmed that IL-1R2^+^TIGIT^+^CD4^+^T subset was markedly increased in both thymus and spleen in CD11c-p28^f/f^ donors than WT mice (Supplementary Fig. 6a, b). Enhanced *Bhlhe40* expression in CD4^+^ T cells has been demonstrated to promote aGVHD pathogenesis.^49^ Other T cell effector genes, including *Nr4a1* and *Btla* were increased, whereas *Ccr7*, *Ccr9*, and *Klf2* were decreased (Supplementary Fig. 7a), suggesting that IL-27 p28 deficiency promotes pro-inflammatory and pathogenic fates of CD4^+^ T cells in the thymus. Pathway analysis performed by gene-set enrichment analysis (GSEA) comparing CD4 SP Cluster 1 with Cluster 0 revealed that the expression patterns of genes known to involve in cytokine production and cytokine-related responses were increased with IL-27 p28 deficiency (Fig. 4j). Signals include response to cytokine, cytokine binding, and cytokine receptor activity were enriched in IL-27 p28-deficient CD4 SP cells compared to WT CD4 SP cells (Supplementary Fig. 8). We further assessed the proportions of IL-1R2^+^TIGIT^+^CD4^+^ T cells in murine aGVHD models and clinical patients with or without aGVHD. Consistent with scRNA-seq, donor-derived IL-1R2^+^TIGIT^+^CD4^+^T were significantly increased in recipients of CD11c-p28^f/f^ grafts compared with those of WT controls (Fig. 4k, l). Moreover, this pathogenic CD4^+^T subset was also increased in grade II-IV severe aGVHD patients compared with non-aGVHD patients after allo-HSCT (Fig. 4m, n). Our results therefore suggest that T cells are indeed defective in their steady-state in CD11c-p28^f/f^ mice, as demonstrated by impaired Treg cell generation and increased IL-1R2^+^TIGIT^+^ pathogenic CD4^+^ T cells in the thymus, which are the main driving force of aGVHD development.

### IL-27 p28 deficiency restrains Treg cell differentiation via the IFN-γ/STAT1 signaling pathway

**Fig. 5:**
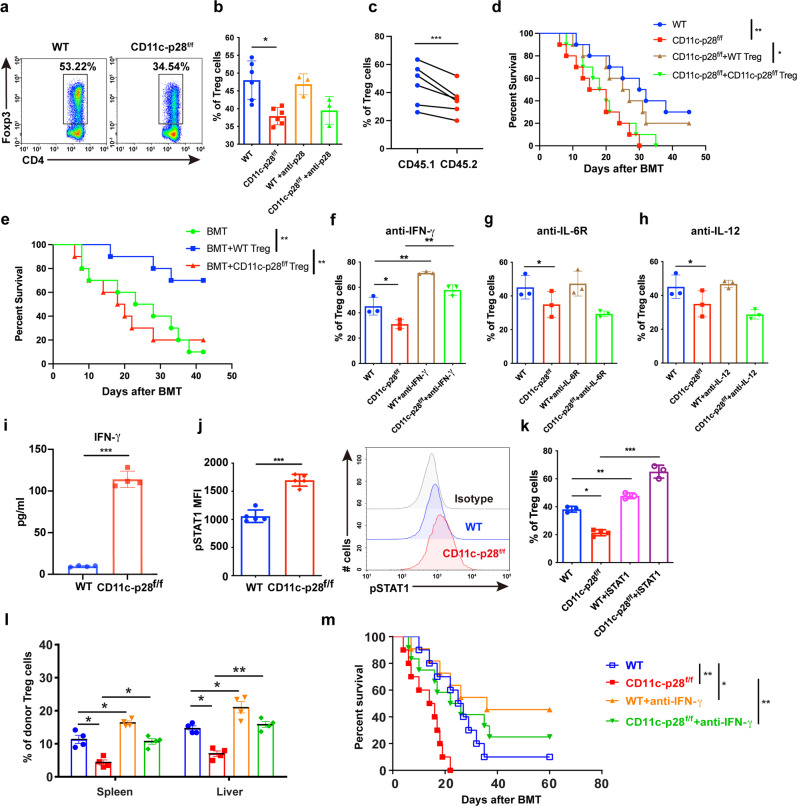
IL-27 p28 deficiency restrains Treg cell differentiation via the IFN-γ/STAT1 signaling pathway. **a**, **b** Splenocytes from WT or CD11c-p28^f/f^ mice were induced for Treg polarization in the presence or absence of IL-27 p28 antibody (10 μg/ml). Representative figures and summary data of the frequency of Tregs are depicted. **c** BALB/c recipients were injected with 1 × 10^7^ TCD-BM cells from CD45.1 mice, along with an equal number of T cells (2 × 10^6^) from CD11c-p28^f/f^ mice and CD45.1 congenic mice. Treg cell reconstitution was evaluated in vivo 14 days post transfer. **d** BALB/c recipients were transplanted with 5 × 10^6^ TCD-BM cells plus 1 × 10^6^ conventional T cells (Tconv) either from WT or CD11c-p28^f/f^ mice and transferred with 7.5 × 10^5^ Treg cells from WT or CD11c-p28^f/f^ mice respectively. The overall survival curve is depicted (*n* = 10 per group). **e** BALB/c recipients were transplanted with 5 × 10^6^ TCD-BM cells plus 1 × 10^6^ Tconv cells from WT donors and transferred with or without 7.5 × 10^5^ Tregs from WT or CD11c-p28^f/f^ mice (*n* = 10 per group). **f**–**h** Anti-IFN-γ, anti-IL-6R, or anti-IL-12 were added during Treg polarization at a concentration of 10 μg/ml. Percentage of Treg cells was detected by FACS (*n* = 3 per group). **i** IFN-γ production in the supernatant of polarized Tregs was detected by ELISA (*n* = 4 per group). **j** The expression of phosphorylated STAT1 among Treg populations is shown (*n* = 5 per group). **k** STAT1 inhibitor (10 μM) was added during Treg polarization. Percentages of Treg cells were detected by FACS (*n* = 3 per group). **l**, **m** BALB/c recipients were transplanted with 1 × 10^7^ WT BMs together with 5 × 10^6^ splenocytes from either WT or CD11c-p28^f/f^ mice. Recipients were injected with αIFN-γ (250 μg per mouse) after BMT. Percentages of Tregs were detected 2 weeks after BMT (*n* = 4 per group). The overall survival curve is depicted (*n* = 10 per group). Data are representative of three independent experiments and presented as mean ± SD. *P < 0.05; **P < 0.01; ***P < 0.001

To further investigate the mechanism by which IL-27 p28 deficiency regulates Treg differentiation, we performed an in vitro Treg cell differentiation assay. We found that Treg cell differentiation was significantly impaired in CD11c-p28^f/f^ mice (Fig. 5a). However, blocking p28 signaling using a p28 neutralizing antibody did not show similar effect (Fig. 5b). To further investigate whether IL-27 p28 regulates Treg cell differentiation in a cell-intrinsic manner, recipients were injected with TCD-BM cells from CD45.1 mice, along with an equal number of T cells from CD11c-p28^f/f^ or CD45.1 congenic mice. Treg cell reconstitution was evaluated in vivo 14 days post transfer. The results showed that Treg cell reconstitution from IL-27 p28-deficient mice was significantly reduced compared to their CD45.1 counterparts (Fig. 5c), indicating a cell-intrinsic impairment of Treg cell development due to IL-27 p28 deficiency. To further evaluate the functions of Treg cells from WT or CD11c-p28^f/f^ mice in controlling of aGVHD, we performed a Treg cell rescue experiment by co-transferring an equal number of Treg cells from WT or CD11c-p28^f/f^ mice, respectively. The survival curve showed that co-transfer of Treg cells from WT mice significantly mitigated aGVHD, while co-transfer of Treg cells from CD11c-p28^f/f^ mice had no protective effect against aGVHD (Fig. 5d). Moreover, Treg cells from CD11c-p28^f/f^ mice also failed to control aGVHD induced by WT T cells, whilst WT Treg cells significantly prolonged the survival of the recipients (Fig. 5e). Therefore, these results suggest that IL-27 p28 deficiency dampened Treg cell functions during aGVHD development.

IFN-γ, IL-6, and IL-12 are cytokines that can inhibit Treg cell differentiation. We demonstrated that blocking IFN-γ, but not IL-6 or IL-12 could restore the inhibition of Treg cell differentiation caused by IL-27 p28 deficiency (Fig. 5f–h). We also observed a substantially elevated IFN-γ production during Treg cell differentiation after IL-27 p28 deficiency (Fig. 5i). However, IL-27 p28 expression was undetectable in the supernatants during Treg cell differentiation in both WT and CD11c-p28^f/f^ mice (Data not shown). These results suggest that T cell-intrinsic IFN-γ signal is responsible for the impaired Treg cell differentiation. STAT1 is a key mediator of IFN-γ signaling that regulates the differentiation of Treg cells.^50^ We observed that phosphorylation of STAT1 was largely increased in Treg cells differentiated from CD11c-p28^f/f^ mice compared with those from WT mice (Fig. 5j). In addition, STAT1 inhibitor added in the culture medium significantly reversed the IL-27 p28-mediated inhibition of Treg cell differentiation (Fig. 5k). Furthermore, blocking IFN-γ signaling in vivo increased Treg cell numbers in the spleen and liver and ameliorated aGVHD in mice receiving CD11c-p28^f/f^ donor cells (Fig. 5l, m), suggesting that IL-27 p28 deficiency inhibits Treg differentiation and function in an IFN-γ-dependent manner.

### IL-27 p28 is a valuable marker for predicting aGVHD after allo-HSCT in humans

**Fig. 6:**
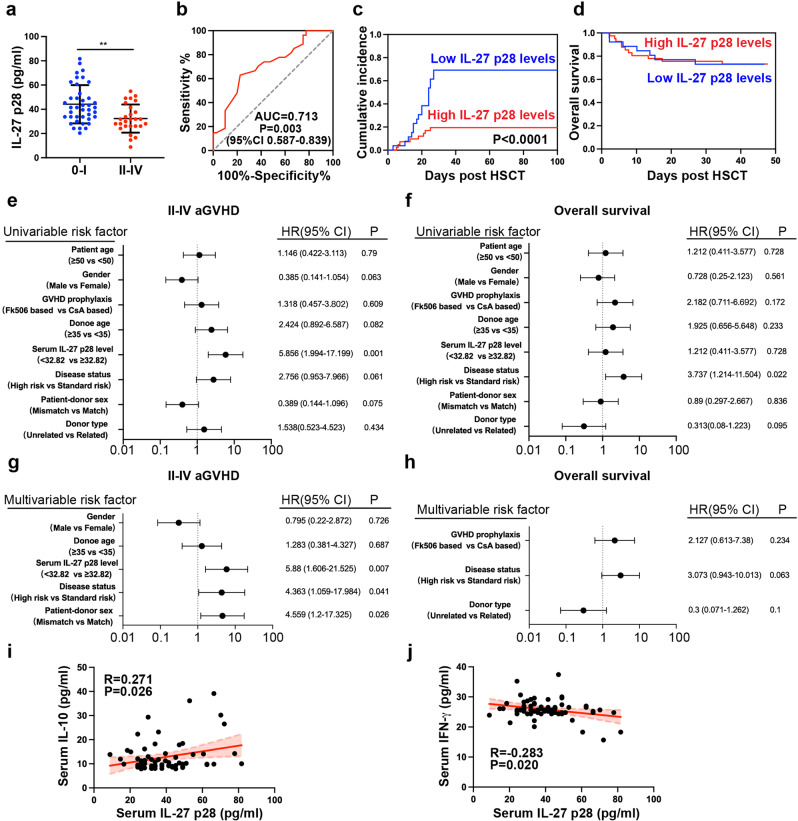
IL-27 p28 is a valuable marker for predicting aGVHD after allo-HSCT in humans. **a** IL-27 p28 production in patients after allo-HSCT were examined by ELISA. **b** ROC curve was constructed to predict severe aGVHD occurrence. **c**, **d** The cumulative incidence of severe aGVHD (**c**) and overall survival (**d**) between patients with high and low IL-27 p28 levels are shown. **e**, **f** Univariate analyses of factors associated with severe aGVHD occurrence (**e**) or overall survival (**f**) after allo-HSCT. **g**, **h** Multivariate analyses of factors associated with severe aGVHD occurrence (**g**) or overall survival (**h**) after allo-HSCT. **i**, **j** The associations between the expression of IL-27 p28 and IL-10 (**i**), and IFN-γ (**j**) were analyzed. Data are presented as mean ± SD. **P < 0.01

Although the effect of IL-27 p28 in murine aGVHD has been reported, the clinical significance of IL-27 p28 in aGVHD patients remains undetermined. Hence, we detected IL-27 p28 expression in the serum of 67 patients who underwent allo-HSCT and found that patients with severe aGVHD (Grade II-IV, *n* = 27) displayed significantly decreased IL-27 p28 levels compared with patients with no/low-grade aGVHD (Grade 0-I, n = 40, *P* = 0.0015, Fig. 6a). When using 32.82 ng/ml as the cutoff value, IL-27 p28 levels could predict the occurrence of severe aGVHD based on ROC analysis (Fig. 6b). The sensitivity and specificity were 77.5% and 63%, respectively. Patients with high levels of IL-27 p28 had a significantly lower incidence of severe aGVHD but showed a similar OS rate compared with patients with low levels of IL-27 p28 (Fig. 6c, d). Consistently, univariate analyses showed that high levels of IL-27 p28 were significantly associated with a low incidence of severe aGVHD but not OS (Fig. 6e, f). High IL-27 p28 and disease status were independent factors for predicting severe aGVHD (HR = 5.88, 95% CI 1.606-21.525, *P* = 0.007, and HR = 4.363, 95% CI 1.059-17.984, *P* = 0.041, Fig. 6g) but not OS (Fig. 6h). IL-27 p28 showed similar levels among different primary diseases or cGVHD development (Supplementary Fig. 9a, b). Similar results were observed by evaluating IL-27 heterodimer in predicting aGVHD (Supplementary Fig. 10). Thus, IL-27 p28 or IL-27 may serve as useful predictors of severe aGVHD. In addition, we found that IL-27 p28 was positively correlated with IL-10 (*r* = 0.271, *P* = 0.026), whereas negatively correlated with IFN-γ (*r* = −0.283, *P* = 0.02; Fig. 6i, j), indicating that serum levels of IL-27 p28 are associated with Treg/Teff cell balance in humans.

## Discussion

The anti-inflammatory roles of IL-27 have been demonstrated in the prevention of inflammation-induced liver injury, cecal ligation, and puncture (CLP)-induced sepsis and infections.^51–53^ Consistent with prior findings, we found that DC-derived IL-27 p28 inhibits allogeneic immune responses and aGVHD development. However, in contrast to our observation in recipients with IL-27 p28^-/-^ grafts as well as CD11c-p28^f/f^ grafts,^25^ blocking p28 signaling using IL-27 p28 antibody reduced GVHD severity and prolonged mice survival.^25,26^ One explanation is that the commercial p28-blocking antibody mainly neutralizes the function of the proinflammatory cytokine IL-27 in vivo,^25^ and this antibody was derived from the sera of mice immunized with a p28-EBI3 fusion protein linked to OVAglu.^54^ Interestingly, p28 antibody treatment could still attenuate GVHD mortality in recipients of IL-27 p28^-/-^ allografts, indicating that this antibody has an additional neutralization function independent of p28.^25^

Using scRNA-seq, we found that T cell development was impaired during thymic selection in CD11c-p28^f/f^ mice. IL-27 p28 deficiency significantly inhibited DP cells and promoted CD4 and CD8 SP T cells. In particular, IL-27 p28 deficiency inhibited Treg cell generation and promoted T cell activation. Transcriptomics results revealed that several critical genes, including *Klf2*, *Bach2*, *Cd122* that orchestrate Treg cell development were reduced in IL-27 p28-deficient Treg cells. A recent study demonstrated that *Klf2* expression could separate Treg maturity. KLF2 positive cells were responsible for T cell migration, leukocyte cell adhesion, and negatively correlated with Th17 cell differentiation as well as cytokine production.^55^ *Bach2* is required for coordinating Treg differentiation and homeostasis.^56^ A previous study revealed that mice lacking CD122 died from lethal T cell-driven autoimmunity due to a reduced number of functional Tregs.^57^ In addition, we observed increased expression of *Tnfrsf9*, *Tight*, *Lag3*, *Pdcd1*, *Batf*, *Maf*, *Bhlhe40*, *Nr4a1*, *Tnfrsf18*, and *Tnfrsf4* in CD11c-p28^f/f^ mice, suggesting that IL-27 p28 deficiency promotes T cell activation and pathogenicity in the thymus. *Nr4a1*, downstream of T cell receptor signal, is involved in T cell early activation by inducing *Tnfrsf9* expression.^58,55^ *Batf* is essential for effector T cells development.^59,60^ The expression of T cell-intrinsic *Batf* induces pathogenic GM-CSF^+^ effector T cell development, which is sufficient to promote aGVHD.^61^ A previous study also showed that reduced IL-7R was associated with increased aGVHD incidence.^62^ T-cell activation was characterized by high expression of these genes, and IL-27 p28 depletion may contribute to aGVHD by regulating the pathogenicity fate of CD4^+^ T cells in the early phase of thymic development.

In contrast to the inhibitory role of IL-27 p28, administration of IL-27 exacerbated aGVHD in mice,^27^ and this effect was dependent on IL-27Rα, suggesting that IL-27 plays a proinflammatory function by signaling through IL-27Rα. IL-27 was reported to promote Th1 cell polarization, inhibit Th17 cell development, whereas promote IL-10 production by CD4^+^ T cells.^12,13,17^ We found that IL-27 p28 deficiency suppressed Treg cell differentiation during aGVHD. It was reported that endogenous IL-27 p28 can partially antagonize gp130-mediated signaling in response to IL-6, IL-11, and IL-27.^63^ However, another study using purified IL-27 p28 protein from bacteria showed that a high dose of p28 could bind to gp130 but exert an agonistic function.^64^ Therefore, IL-27 p28 may bind to unknown receptors in vivo to exert its anti-inflammatory function.

Although the potential role of IL-27 and IL-27R in aGVHD development has been studied, the clinical significance of IL-27 p28 in this context was largely unappreciated. Consistent with our results in murine models, patients with high serum levels of IL-27 p28 had a significantly decreased incidence of severe aGVHD. High serum levels of IL-27 p28 can serve as an independent biomarker for predicting severe aGVHD in patients who underwent allo-HSCT. However, our findings are a correlation conclusion but could not demonstrate the causal relationship between IL-27 p28 and aGVHD in human settings. A prior study showed that artificially secreted human IL-27 p28 has an immunoregulatory effect.^65^ Therefore, future studies are needed to investigate the role of human IL-27 p28 in aGVHD using appropriate xenograft models.

In conclusion, our findings reveal an intrinsic role of DC-derived IL-27 p28 in the pathogenesis of aGVHD in mice. IL-27 p28 regulates T cell program in pathogenicity during T cell development and in the setting of aGVHD. In addition, serum IL-27 maybe a valuable prognostic biomarker in predicting the incidence of moderate-to-severe aGVHD in patients underwent allo-HSCT. Our results provide a mechanistic rationale for efforts to modulate Treg/Teff cell balance and prevent aGVHD by regulating IL-27 p28.

## Materials and methods

### Mice and patient samples

C57BL/6, BALB/c mice, and CD45.1 mice (B6 background) were obtained from Vital River Laboratory (Beijing, China). IL-27p28^flox/flox^-CD11c-cre (CD11c-p28^f/f^) mice were kindly provided by Dr. Zhinan Yin.^35^ IL-27 p28^flox/flox^ mice were used as WT controls. All mice were maintained in the specific pathogen-free facilities of Soochow University. Serum samples at the time of preconditioning from 67 patients who underwent allo-HSCT were recruited in our previous study.^66^ Informations are shown in Supplementary Tables 1 and 2. For clinical flow cytometry analysis, we collected peripheral blood of 3 healthy donors and 17 patients with written informed consent after allo-HSCT. All protocols were approved by the Ethics Committee of Soochow University.

### Establishment of the aGVHD model and histology assessment

BALB/c mice were used to establish murine aGVHD model as our previous works otherwise specified.^4,66,67^ Briefly, lethally irradiated BALB/c recipients (650 cGy, X-Ray) were intravenously injected 1 × 10^7^ bone marrow (BM) cells together with 5 × 10^6^ splenocytes (SPs) from BALB/c, C57BL/6, and CD11c-p28^f/f^ mice. BMDCs were cultured with GM-CSF and IL-4 (10 ng/ml) for 7 days from donors. Recipients have injected with 5 × 10^6^ TCD-BMs and 1 × 10^6^ T cells from WT mice together with either 1 × 10^6^ WT or CD11c-p28^f/f^ DCs at day 0, 1, and 2 post-BMT. In some experiments, recipients received either WT or CD11c-p28^f/f^ allografts of 5 × 10^6^ TCD-BMs and 1 × 10^6^ T cells as indicated. For Treg rescue assay, recipients have infused of 5 × 10^6^ TCD-BM cells plus 1 × 10^6^ conventional T cells (Tconv) together with or without 7.5 × 10^5^ Treg cells either from WT or CD11c-p28^f/f^ mice, respectively. To compare naive T cell functions, recipients received 5 × 10^6^ TCD-BMs from WT mice together with 1 × 10^6^ naive T cells from WT or CD11c-p28^f/f^ mice, respectively. TCD-BMs, splenic T cells and naive T cells were isolated by Mouse CD90.2 Positive Selection Kit, T Cell Isolation Kit and Pan-Naive T Cell Isolation Kit (Stem Cell, Canada). The purity of naive T cells was more than 95% as confined by staining of surface markers including CD62L, CD44, and CD3 (Supplementary Fig. 11). For the host IL-27 p28 deficiency aGVHD model, C57BL/6 or CD11c-p28^f/f^ recipients received lethal irradiation (800 cGy) and were transplanted with 1 × 10^7^ BMs and 7.5 × 10^7^ splenocytes from BALB/c mice. Anti-IFN-γ or control IgG1 antibody (BioXCell, West Lebanon, NH) was injected at 250 μg per mouse on days −1, 6, and 13 for IFN-γ blocking as previously described.^66^ Representative samples of aGVHD tissue were obtained 2 weeks after transplantation for histology assessment as previously reported.^66,67^

### Mixed lymphocyte reaction (MLR)

BMDCs were induced as mentioned above. A total of 1 × 10^4^ irradiated DCs were cocultured with 1 × 10^5^ allogeneic T cells from WT or CD11c-p28^f/f^ mice for 5 days. Cell proliferation were detected by a 3H-TdR or CFSE (Invitrogen, Life Technologies, USA) method.

### Flow cytometry

Single-cell suspensions were performed as described previously.^66,67^ Antibodies were listed in Supplementary Table 4 and added for cellular staining at 4 °C for 30 min. Intracellular cytokines and pSTAT1 in induced Treg cells were detected by CytoFix/CytoPerm buffer and Phosflow^TM^ Fix/Perm kit, respectively (BD Biosciences, San Diego, CA). Treg cells were assessed by a FoxP3 staining Kit (eBioscience, San Diego, CA). Data were obtained and analyzed on NovoCyte and NovoExpress software (ACEA Biosciences, San Diego, CA).

### Single-cell RNA capture, sequencing, and processing

The single-cell lysates of thymuses from C57BL/6 and CD11c-p28^f/f^ mice were loaded per channel onto a Chromium^TM^ controller (10x Genomics) after lysis of red blood cells, to generate gel-bead-in-emulsions (GEMs). High-throughput sequencing of the library was performed using the paired-end sequencing model of the Illumina sequencing platform at LC Bio (Zhejiang, China). Data were obtained from CellRanger Version 4.0.0 against Mus_musculus. GRCm38.96 served as the reference genome and then normalized using the Seurat “LogNormalize” function. Principal component analysis (PCA) dimensionality reduction was performed using “RunPCA” after quality control. A resolution parameter of 0.4 was used for the samples. Differential gene expression (DEG) were filtered using a maximum adjusted *P*-value of 0.05 for Gene Ontology analysis as previously described (Supplementary Table 3).^68^ scRNA-seq plots were generated using ggplot2 (v3.3.5), and volcano maps were generated using the EnhancedVolcano (v 1.10.0) package in R 4.0.0. Violin maps and heat maps were generated using the “Vlnplot” and “Doheatmap” functions in Seurat.

### Treg differentiation and suppression assay

Splenocytes or naive CD4^+^ T cells from C57BL/6 and CD11c-p28^f/f^ mice were induced for Treg polarization for 3 days as previously demonstrated.^66^ CD4^+^CD25^−^ effector T (Teff) and Tregs were isolated from splenocytes of C57BL/6 and CD11c-p28^f/f^ mice using microbeads (Miltenyi Biotec, Auburn, CA). Teff cells were labeled with CellTrace CFSE (5 μM, Invitrogen, Carlsbad, CA) and cocultured with C57BL/6 and CD11c-p28^f/f^ Treg cells at the indicated ratios in pre-coated plates for 3 days. The suppressive activity was measured by the proliferation of Teff cells. In some experiments, specific antibodies (10 μg/ml) were added for in vitro blocking.

### Cytokine analysis

Inflammatory cytokines in the serum of aGVHD recipients were assessed by LEGENDplex™ T helper cytokines kit and analyzed with LEGENDplex Data Analysis Software Version 7.1 (Biolegend, San Diego, CA). Mouse IL-27 p28, IL-27, IFN-γ (Biolegend, San Diego, CA), human IL-27 p28 (Abcam, Cambridge, UK), human IL-27, IL-10 and IFN-γ (R&D Systems, Minneapolis, MN) were detected by commercial ELISA kits.

### Statistical analysis

Log-rank test was used to analysis survival of recipients post allo-HSCT. Unpaired Student’s test were performed to comparisons of two means. Correlations between IL-27 p28 levels and aGVHD grade were detected by Spearman’s coefficient. Correlations between IL-27/IL-27p28 and clinical characters were detected by the Mann–Whitney *U* test and Fisher’s exact test. ROC curves were generated to predict aGVHD occurrence. The cutoff value determined from the ROC curves was used to analysis the sensitivity and specificity. aGVHD incidence was detected by Gray’s test. *P* < 0.05 were considered as statistical difference.

## Supplementary information

Supplementary Materials

## Data Availability

All data are available from the corresponding author on reasonable request.

## References

[1] Gooley TA (2010). Reduced mortality after allogeneic hematopoietic-cell transplantation. N. Engl. J. Med..

[2] Ghimire S (2017). Pathophysiology of GvHD and other HSCT-related major complications. Front. Immunol..

[3] Markey KA, MacDonald KP, Hill GR (2014). The biology of graft-versus-host disease: experimental systems instructing clinical practice. Blood.

[4] Cai Y (2018). Adoptively transferred donor IL-17-producing CD4(+) T cells augment, but IL-17 alleviates, acute graft-versus-host disease. Cell Mol. Immunol..

[5] Kumar S, Mohammadpour H, Cao X (2017). Targeting cytokines in GVHD therapy. J. Immunol. Res. Ther..

[6] Liu Y (2015). IL-35 mitigates murine acute graft-versus-host disease with retention of graft-versus-leukemia effects. Leukemia.

[7] Yoshida H, Hunter CA (2015). The immunobiology of interleukin-27. Annu. Rev. Immunol..

[8] Tait Wojno ED, Hunter CA, Stumhofer JS (2019). The immunobiology of the interleukin-12 family: room for discovery. Immunity.

[9] Diegelmann J, Olszak T, Goke B, Blumberg RS, Brand S (2012). A novel role for interleukin-27 (IL-27) as mediator of intestinal epithelial barrier protection mediated via differential signal transducer and activator of transcription (STAT) protein signaling and induction of antibacterial and anti-inflammatory proteins. J. Biol. Chem..

[10] He H (2020). Aging-induced IL27Ra signaling impairs hematopoietic stem cells. Blood.

[11] Jones SA, Jenkins BJ (2018). Recent insights into targeting the IL-6 cytokine family in inflammatory diseases and cancer. Nat. Rev. Immunol..

[12] Pflanz S (2002). IL-27, a heterodimeric cytokine composed of EBI3 and p28 protein, induces proliferation of naive CD4+ T cells. Immunity.

[13] Owaki T (2005). A role for IL-27 in early regulation of Th1 differentiation. J. Immunol..

[14] Yoshida H (2001). WSX-1 is required for the initiation of Th1 responses and resistance to L. major infection. Immunity.

[15] Kilgore AM (2018). IL-27p28 production by XCR1(+) dendritic cells and monocytes effectively predicts adjuvant-elicited CD8(+) T cell responses. Immunohorizons.

[16] Awasthi A (2007). A dominant function for interleukin 27 in generating interleukin 10-producing anti-inflammatory T cells. Nat. Immunol..

[17] Pot C, Apetoh L, Awasthi A, Kuchroo VK (2011). Induction of regulatory Tr1 cells and inhibition of T(H)17 cells by IL-27. Semin Immunol..

[18] Zhang H (2020). An IL-27-driven transcriptional network identifies regulators of IL-10 expression across T helper cell subsets. Cell Rep..

[19] Fitzgerald DC (2007). Suppression of autoimmune inflammation of the central nervous system by interleukin 10 secreted by interleukin 27-stimulated T cells. Nat. Immunol..

[20] Batten M (2006). Interleukin 27 limits autoimmune encephalomyelitis by suppressing the development of interleukin 17-producing T cells. Nat. Immunol..

[21] Do J (2017). Treg-specific IL-27Ralpha deletion uncovers a key role for IL-27 in Treg function to control autoimmunity. Proc. Natl Acad. Sci. USA.

[22] Choi JK (2021). IL-27-producing B-1a cells suppress neuroinflammation and CNS autoimmune diseases. Proc. Natl Acad. Sci. USA.

[23] Kimura D (2016). Interleukin-27-producing CD4(+) T cells regulate protective. Immun. Malar. Parasite Infect. Immun..

[24] Wang Q (2021). IL-27 signalling promotes adipocyte thermogenesis and energy expenditure. Nature.

[25] Belle L (2016). Blockade of interleukin-27 signaling reduces GVHD in mice by augmenting Treg reconstitution and stabilizing Foxp3 expression. Blood.

[26] Marillier RG, Uyttenhove C, Goriely S, Marbaix E, Van Snick J (2014). IL-27p28 is essential for parent-to-F1 acute graft-versus-host disease. Eur. J. Immunol..

[27] Bastian D (2018). IL-27 receptor signaling on T cells augments GVHD severity through enhancing Th1 responses. J. Immunol. Res. Ther..

[28] Daenthanasanmak A (2019). Targeting Sirt-1 controls GVHD by inhibiting T-cell allo-response and promoting Treg stability in mice. Blood.

[29] Blazar BR, MacDonald KPA, Hill GR (2018). Immune regulatory cell infusion for graft-versus-host disease prevention and therapy. Blood.

[30] Zhu J (2018). IL-27 gene therapy induces depletion of Tregs and enhances the efficacy of cancer immunotherapy. JCI Insight.

[31] Cox JH (2011). IL-27 promotes T cell-dependent colitis through multiple mechanisms. J. Exp. Med..

[32] Nguyen QT (2019). IL-27 targets Foxp3^+^ Tregs to mediate antiinflammatory functions during experimental allergic airway inflammation. JCI Insight.

[33] Hall AO (2012). The cytokines interleukin 27 and interferon-gamma promote distinct Treg cell populations required to limit infection-induced pathology. Immunity.

[34] Le HT (2020). Interleukin-27 enforces regulatory T cell functions to prevent graft-versus-host disease. Front. Immunol..

[35] Zhang S (2013). High susceptibility to liver injury in IL-27 p28 conditional knockout mice involves intrinsic interferon-gamma dysregulation of CD4+ T cells. Hepatology.

[36] Heinrichs J (2016). Regulatory T-cell therapy for graft-versus-host disease. J. Immunol. Res. Ther..

[37] Stumhofer JS (2007). Interleukins 27 and 6 induce STAT3-mediated T cell production of interleukin 10. Nat. Immunol..

[38] Giardino G (2020). T-cell immunodeficiencies with congenital alterations of thymic development: genes implicated and differential immunological and clinical features. Front. Immunol..

[39] Tang H (2016). Thymic DCs derived IL-27 regulates the final maturation of CD4(+) SP thymocytes. Sci. Rep..

[40] Hu Z (2017). CCR7 modulates the generation of thymic regulatory T cells by altering the composition of the thymic dendritic cell compartment. Cell Rep..

[41] Nunez NG (2020). Tumor invasion in draining lymph nodes is associated with Treg accumulation in breast cancer patients. Nat. Commun..

[42] Drashansky TT (2019). Bcl11b prevents fatal autoimmunity by promoting Treg cell program and constraining innate lineages in Treg cells. Sci. Adv..

[43] Kitagawa Y (2017). Guidance of regulatory T cell development by Satb1-dependent super-enhancer establishment. Nat. Immunol..

[44] Miyazaki M (2014). Id2 and Id3 maintain the regulatory T cell pool to suppress inflammatory disease. Nat. Immunol..

[45] Yang BH (2019). TCF1 and LEF1 control Treg competitive survival and Tfr development to prevent autoimmune diseases. Cell Rep..

[46] Miragaia RJ (2019). Single-cell transcriptomics of regulatory T cells reveals trajectories of tissue adaptation. Immunity.

[47] Roychoudhuri R (2013). BACH2 represses effector programs to stabilize T(reg)-mediated immune homeostasis. Nature.

[48] Zheng C (2017). Landscape of infiltrating T cells in liver cancer revealed by single-cell sequencing. Cell.

[49] Piper C (2020). Pathogenic Bhlhe40+ GM-CSF+ CD4+ T cells promote indirect alloantigen presentation in the GI tract during GVHD. Blood.

[50] Chang JH, Kim YJ, Han SH, Kang CY (2009). IFN-gamma-STAT1 signal regulates the differentiation of inducible Treg: potential role for ROS-mediated apoptosis. Eur. J. Immunol..

[51] Yan J (2016). Interleukin-30 (IL27p28) alleviates experimental sepsis by modulating cytokine profile in NKT cells. J. Hepatol..

[52] Cao J (2014). IL-27 controls sepsis-induced impairment of lung antibacterial host defence. Thorax.

[53] Montes de Oca M (2020). IL-27 signalling regulates glycolysis in Th1 cells to limit immunopathology during infection. PLoS Pathog..

[54] Uyttenhove C (2011). Amine-reactive OVA multimers for auto-vaccination against cytokines and other mediators: perspectives illustrated for GCP-2 in L. major infection. J. Leukoc. Biol..

[55] Osman A (2021). TCF-1 controls Treg cell functions that regulate inflammation, CD8(+) T cell cytotoxicity and severity of colon cancer. Nat. Immunol..

[56] Zhang H (2021). Bach2 attenuates IL-2R signaling to control Treg homeostasis and Tfr development. Cell Rep..

[57] Suzuki H (1995). Deregulated T cell activation and autoimmunity in mice lacking interleukin-2 receptor beta. Science.

[58] Fassett MS, Jiang W, D’Alise AM, Mathis D, Benoist C (2012). Nuclear receptor Nr4a1 modulates both regulatory T-cell (Treg) differentiation and clonal deletion. Proc. Natl Acad. Sci. USA.

[59] Li P (2012). BATF-JUN is critical for IRF4-mediated transcription in T cells. Nature.

[60] Kurachi M (2014). The transcription factor BATF operates as an essential differentiation checkpoint in early effector CD8+ T cells. Nat. Immunol..

[61] Ullrich E (2018). BATF-dependent IL-7RhiGM-CSF+ T cells control intestinal graft-versus-host disease. J. Clin. Invest..

[62] Kielsen K (2018). Soluble Interleukin-7 receptor levels and risk of acute graft-versus-host disease after allogeneic haematopoietic stem cell transplantation. Clin. Immunol..

[63] Stumhofer JS (2010). A role for IL-27p28 as an antagonist of gp130-mediated signaling. Nat. Immunol..

[64] Garbers C (2013). An interleukin-6 receptor-dependent molecular switch mediates signal transduction of the IL-27 cytokine subunit p28 (IL-30) via a gp130 protein receptor homodimer. J. Biol. Chem..

[65] Muller SI (2019). A folding switch regulates interleukin 27 biogenesis and secretion of its alpha-subunit as a cytokine. Proc. Natl Acad. Sci. USA.

[66] Gong H (2018). IL-17C mitigates murine acute graft-vs.-host disease by promoting intestinal barrier functions and treg differentiation. Front. Immunol..

[67] Han J (2017). Inhibition of acute graft-versus-host disease with retention of graft-versus-tumor effects by dimethyl fumarate. Front. Immunol..

[68] Tong X (2022). Fucosylation promotes cytolytic function and accumulation of NK cells in B cell lymphoma. Front. Immunol..

