# Supplementary material for: Dendritic cell-derived IL-27 p28 regulates T cell program in pathogenicity and alleviates acute graft-versus-host disease
Source: Signal Transduct Target Ther. 2022 Sep 16;7:319. doi: 10.1038/s41392-022-01147-z (PMC9477797; doi:10.1038/s41392-022-01147-z)
Supplement: Supplementary file 1 — Supplementary Materials [file 41392_2022_1147_MOESM1_ESM.docx]

Supplementary Materials for

Dendritic cell-derived IL-27 p28 regulates T cell program in pathogenicity and alleviates acute graft-*versus*-host disease

Huanle Gong^1, 2, 8^, Shoubao Ma^1, 2, 8, *^, Jia Chen^1, 2, 8^, Bingyu Yang^1, 2, 3, 8^, Shuangzhu Liu^1, 2, 8^, Xin Liu^1^, Jingjing Han^1, 2^, Xiaojin Wu^1, 2^, Lei Lei^1, 2^, Zhinan Yin^4^, Hongjian Sun^5^, Di Yu^6^, Haiyan Liu^7,*^ , Yang Xu ^1, 2, *^, Depei Wu ^1, 2, *^

Correspondence to: Shoubao Ma, [mashoubao@suda.edu.cn](mailto:mashoubao@suda.edu.cn); Haiyan Liu, [micliuh@nus.edu.sg](mailto:micliuh@nus.edu.sg); Yang Xu, [yangxu@suda.edu.cn](mailto:yangxu@suda.edu.cn); Depei Wu, [drwudepei@163.com](mailto:drwudepei@163.com)

**This PDF file includes:**

Figures. S1 to S11

Tables. S1 to S4

**Fig. S1.**


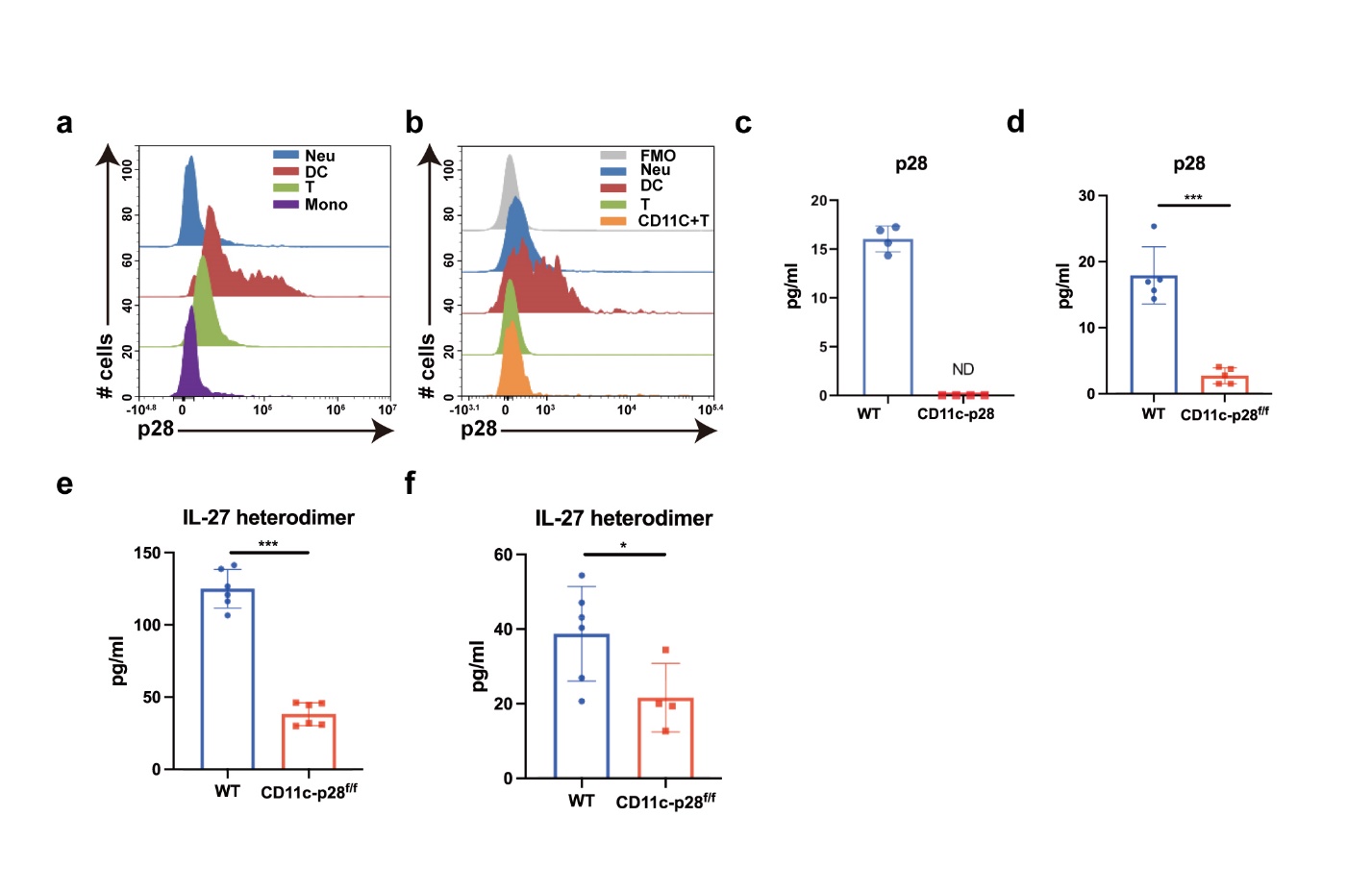


**Fig. S1 DCs are the main source of IL-27 p28 in aGVHD. a** Peripheral blood mononuclear cells from aGVHD patients were stained for IL-27 p28 expression in the indicated cell subsets by FACS. **b** Lethally irradiated BALB/c recipients were transplanted with 1×10^7 BM cells and 5 ×10^6 splenocytes from WT mice. IL-27 p28 expression were detected in neutrophils, DCs, CD3^+^ T, CD11c^+^CD3^+^ T 7 days post BMT. **c** IL-27 p28 expression in serums of WT and CD11c-p28^f/f^ mice was shown as a baseline (n=4 per group). **d** IL-27 p28 expression in serums of recipients with WT and CD11c-p28^f/f^ allografts were detected by ELISA 7 days post-BMT (n=5 per group). **e** The baseline of IL-27 heterodimer expression was shown as in **(c)** (n=4 per group). **f** Expression of IL-27 heterodimer was shown as in **(d)** (n=5 per group). Data are representative of two independent experiments and presented as mean ± SD. ***, P < 0.001.

DCs are the main source of IL-27 p28 in aGVHD.

**Fig. S2.**

**
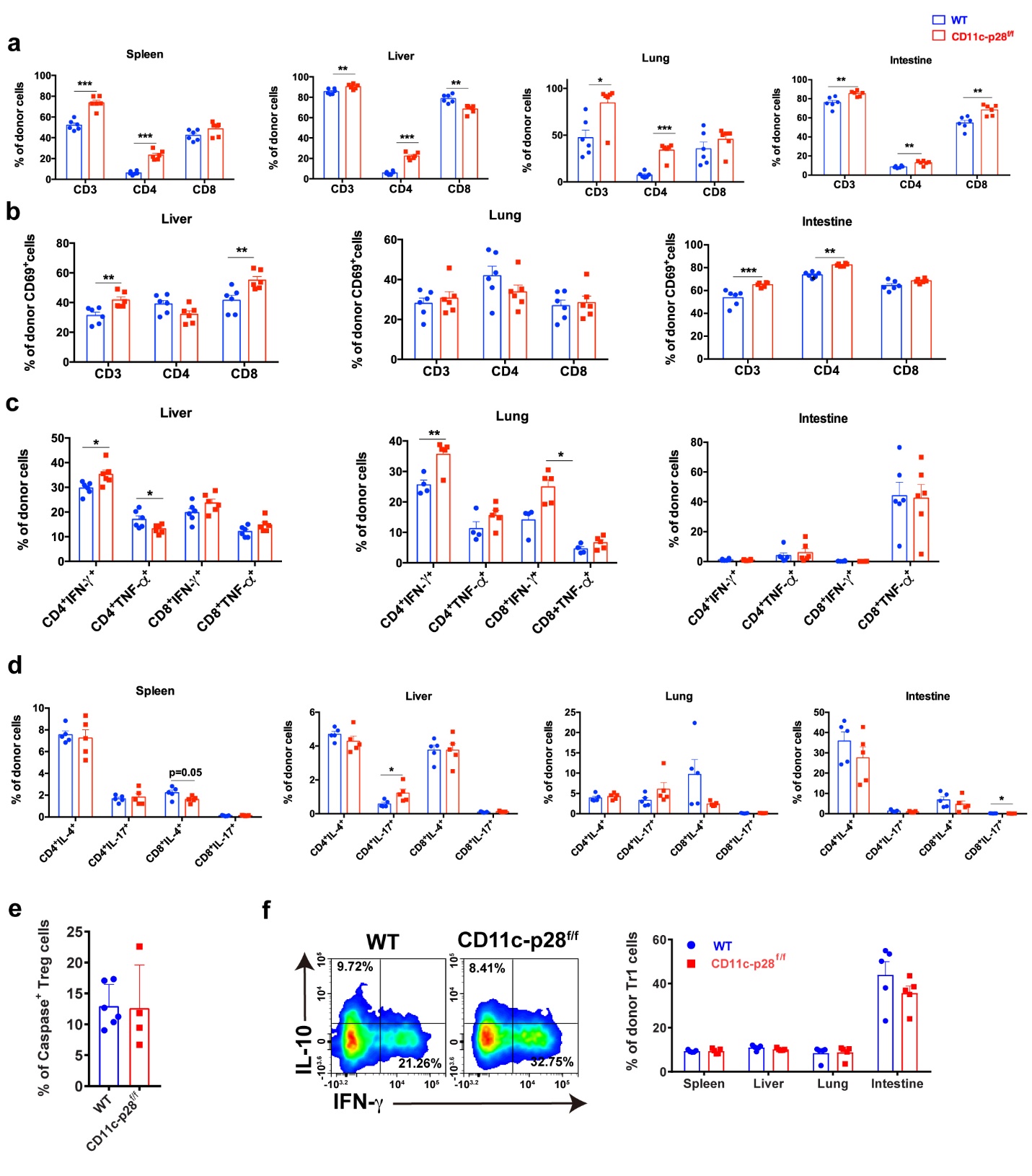
**

**Fig. S2 IL-27 p28 deficiency promotes pathogenic T cells in aGVHD tissues. a-d** Lethally irradiated BALB/c recipients were transplanted with 1×10^7 BM cells from WT mice together with 5 ×10^6 splenocytes from either WT mice or CD11c-p28^f/f^ mice. Immune cell subsets were examined 14 days post-transplantation. Summary data of **(a)** donor CD3^+^ T, CD4^+^ T and CD8^+^ T cells, **(b)** activated CD3^+^ T, CD4^+^ T and CD8^+^ T cells, **(c)** IFN-γ- or TNF-α-producing CD4^+^ T and CD8^+^ T cells, **(d)** IL-4- or IL-17-producing CD4^+^ T and CD8^+^ T cells in the spleen, liver, lung, and intestine are shown (n = 4-6 per group). **e** The frequency of active caspase 3 expression on Treg cells is shown (n = 4-6 per group). **f** Representative plots and summary data of the frequency of IL-10 expression by CD4^+^ T cells (n = 4-6 per group). Data are representative of three independent experiments and presented as mean ± SD. *, P < 0.05; **, P < 0.01; ***, P < 0.001.

**Fig. S3.**

**
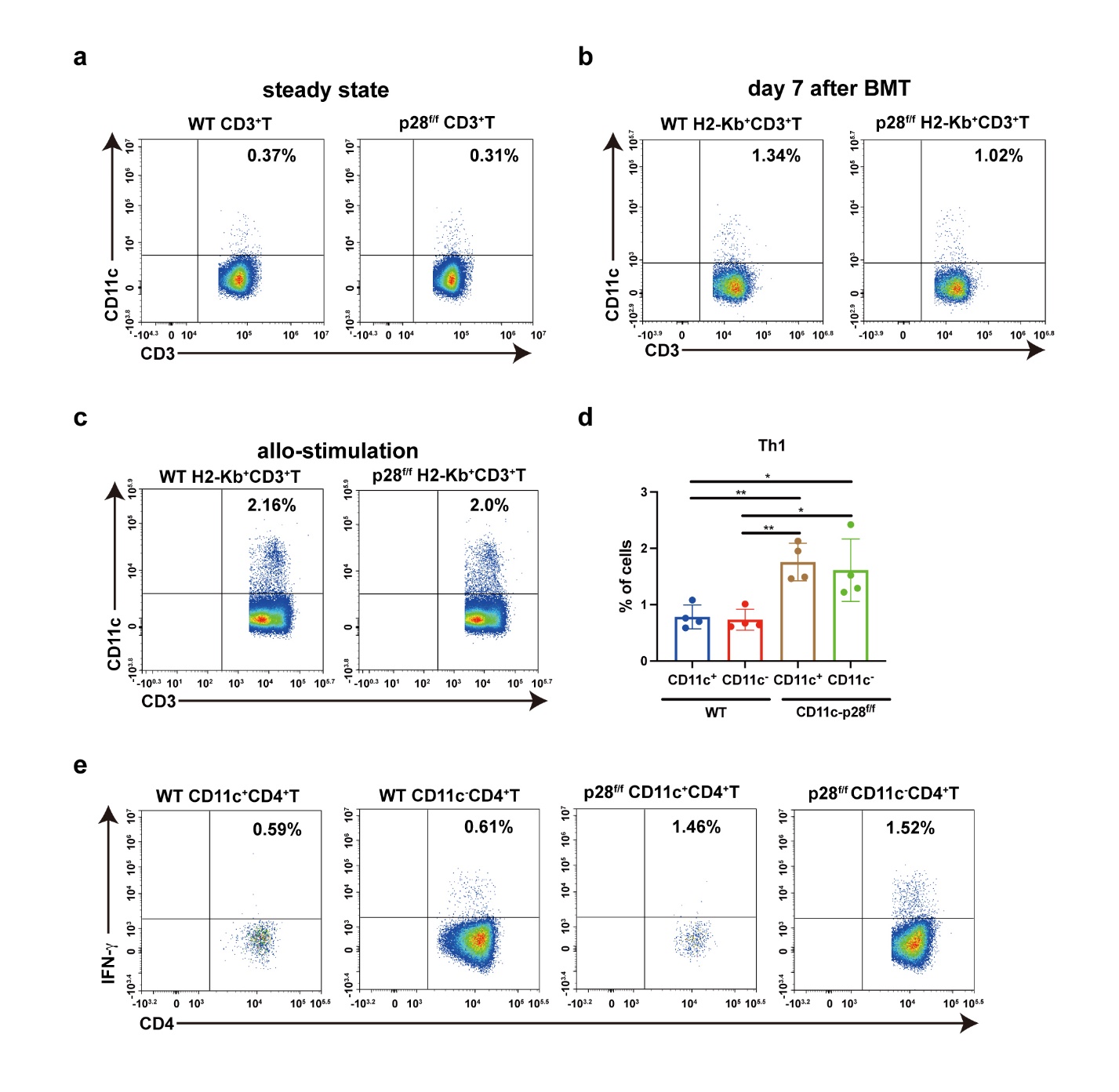
**

**Fig. S3 IL-27 p28 deficiency didn’t affect CD11c^+^ T cell function. a** CD11c expression levels on CD3^+^T cells in WT and CD11c-p28^f/f^ mice at steady states were assessed. **b** CD11c expression levels on donor-derived T cells were detected 7 days post BMT. **c** T cells from WT and CD11c-p28^f/f^ mice were cocultured with BALB/c DCs for 5 days. CD11c expression on T cells from WT or CD11c-p28^f/f^ mice was analyzed. Representative plots were shown. **d, e** Summary data and representative plots of the frequency of IFN-γ production by CD4^+^ T cells from WT and CD11c-p28^f/f^ mice were detected as in **(c)** (n = 4 per group). Data are representative of two independent experiments and presented as mean ± SD. *, P < 0.05; **, P < 0.01.

**Fig. S4.**


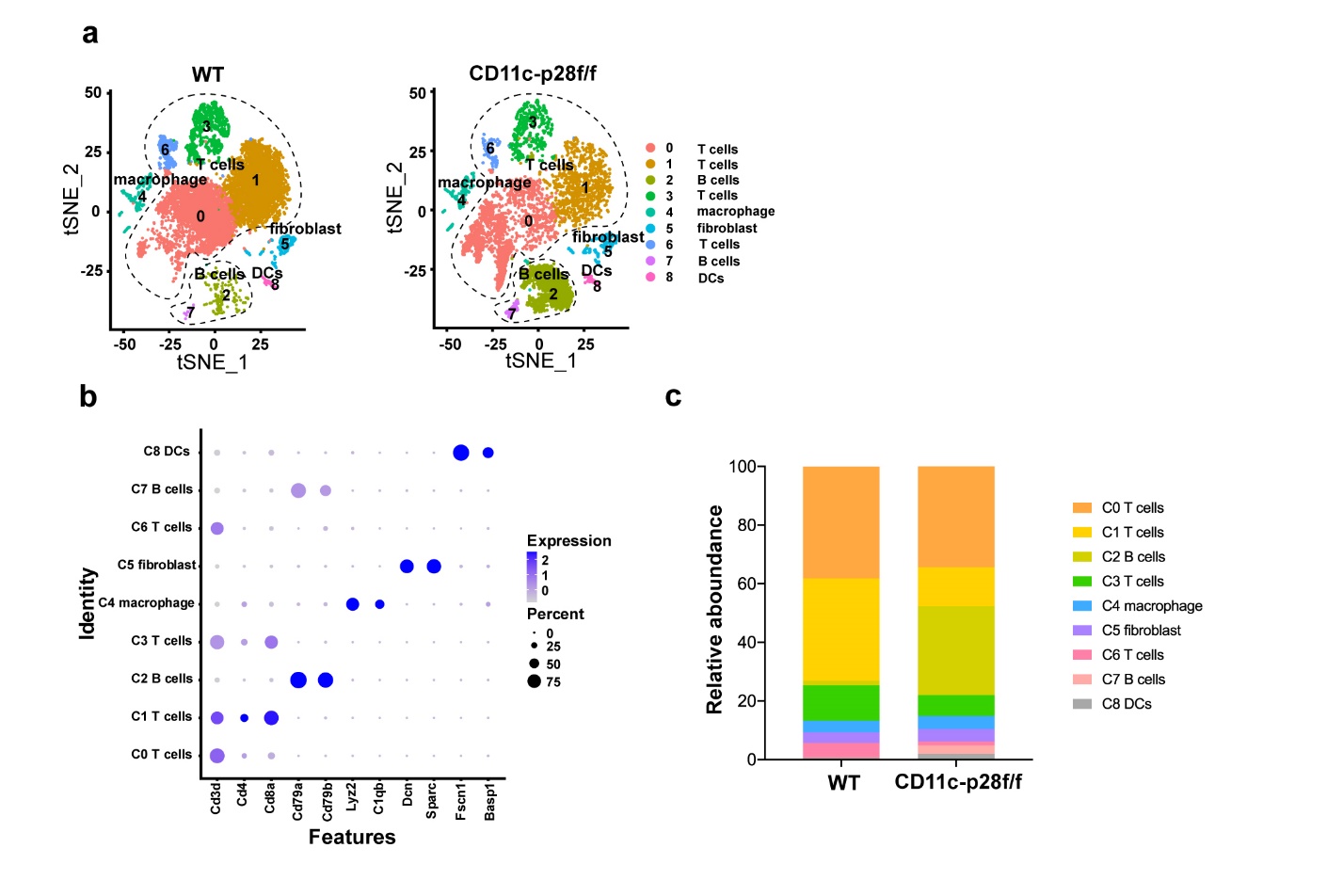


**Fig. S4** **Clusters of thymic cells were assessed via scRNA-seq in WT and CD11c-p28^f/f^ mice. a** Two-dimensional t-SNE visualization of identified cell subpopulations of thymus from CD11c-p28^f/f^ or WT mice. **b** Heatmap of different markers in each of the 9 clusters defined by scRNA-seq analysis. Color depicts the enrichment significance and the dot size depicts the enrichment fold. **c** Relative abundance of different clusters in each sample.

**Fig. S5.**


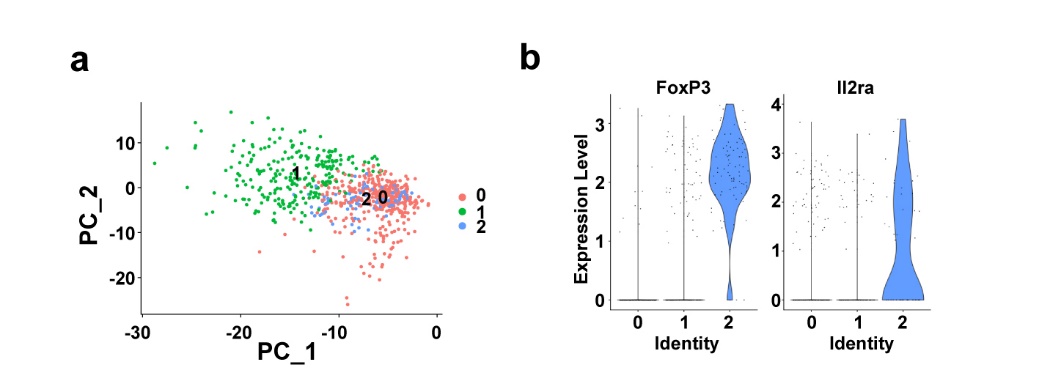


**Fig. S5 Annotation of CD4 SP subclusters.** **a** PCA of CD4 SP subclusters. **b** Relative expression of Treg signature genes Foxp3 and Il2ra in CD4 SP subclusters.

**Fig. S6.**

**
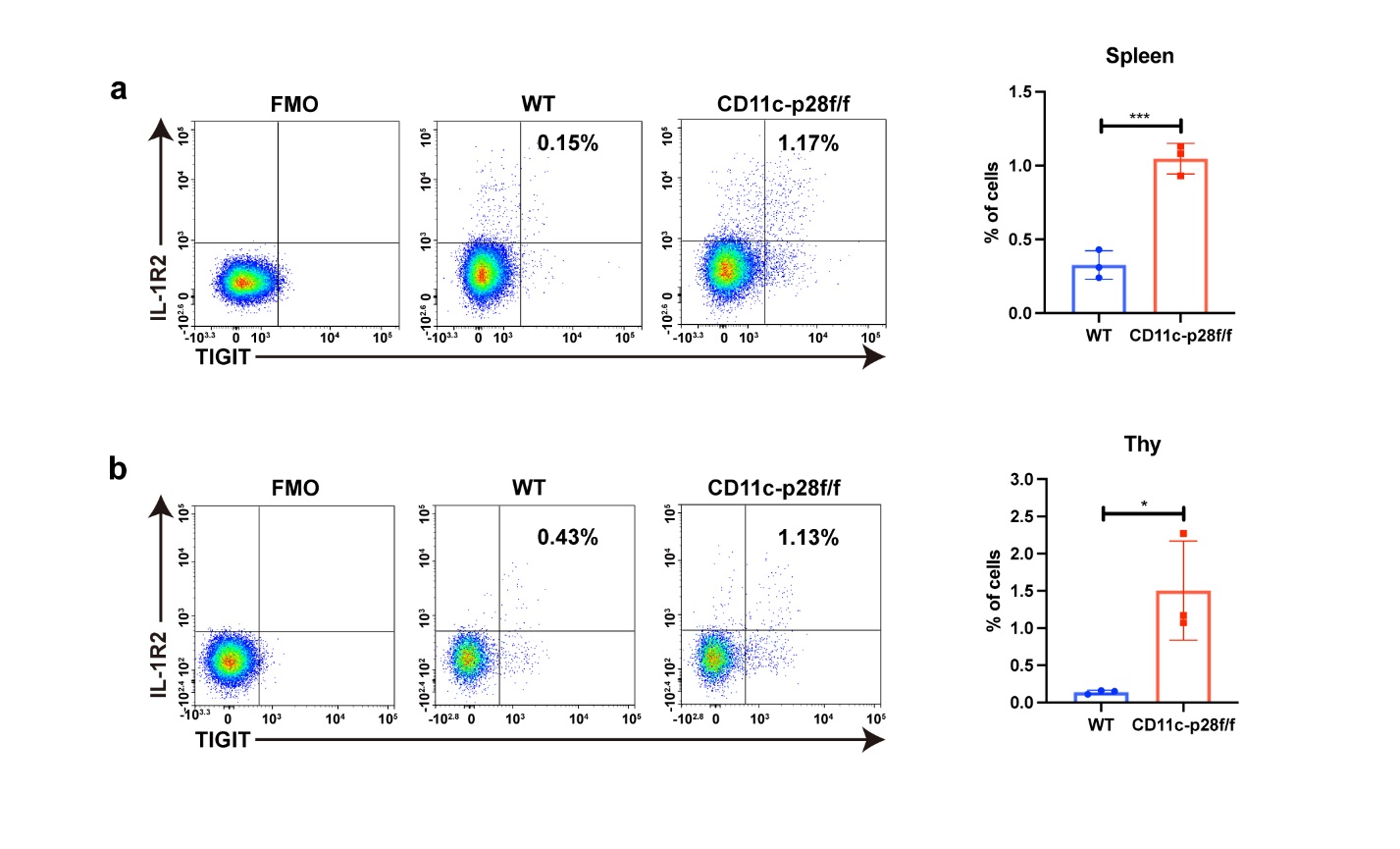
**

**Fig. S6 Proportions of IL-1R2^+^TIGIT^+^CD4^+^T cells in donor mice. a, b** Percentage of IL-1R2^+^TIGIT^+^CD4^+^T cells in splenocytes **(a)** and thymus **(b)** of WT and CD11c-p28^f/f^ mice were detected by FACS (n = 3 per group). Representative figures and summary data of the frequency are depicted. Data are presented as mean ± SD.*, P < 0.05; **, P < 0.01; ***, P < 0.001.

**Fig. S7.**


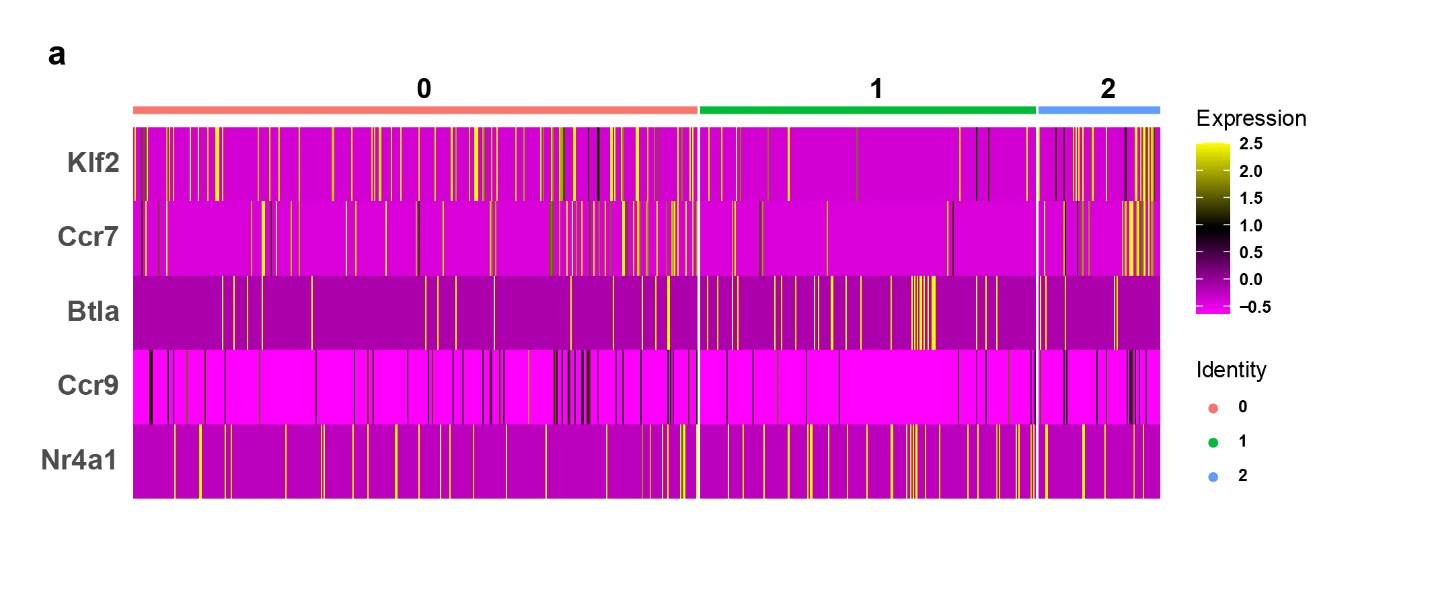


**Fig. S7** **Differential gene expression in CD4 SP subclusters.** **a** Heatmap of CD4 SP cells with 3 main clusters identified. The selective functional-related genes are marked.

**Fig. S8.**


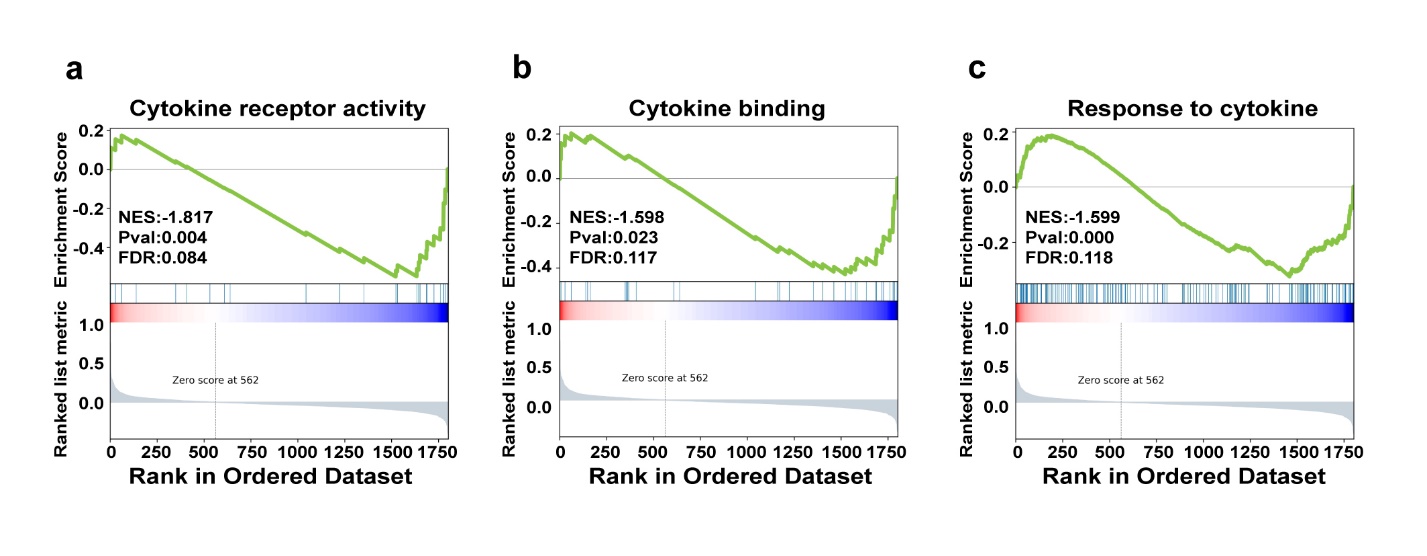


**Fig. S8** **GSEA of CD4 SP cells.** **a-c** GSEA plots showing the enrichment of signals in CD4 SP cells from CD11c-p28^f/f^ mice relative to those from WT mice.

**Fig. S9.**


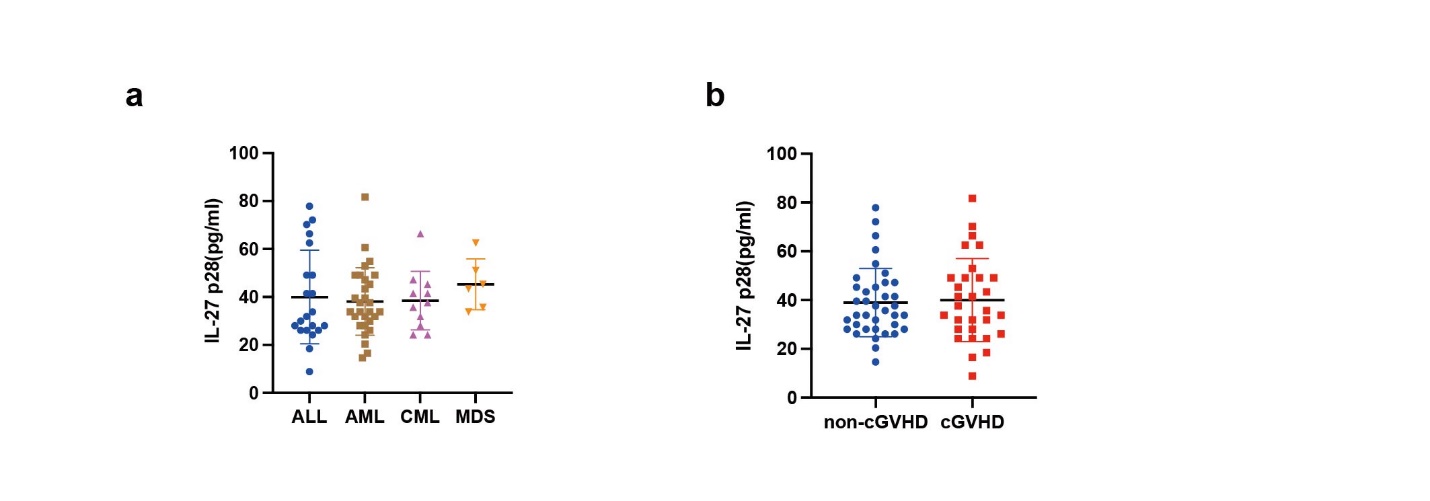


**Fig. S9 Serum IL-27 p28 expressions among different patients.** **a** Serum IL-27 p28 levels among patients with different primary diseases. **b** Serum IL-27 p28 levels in patients with or without cGVHD.

**Fig. S10.**


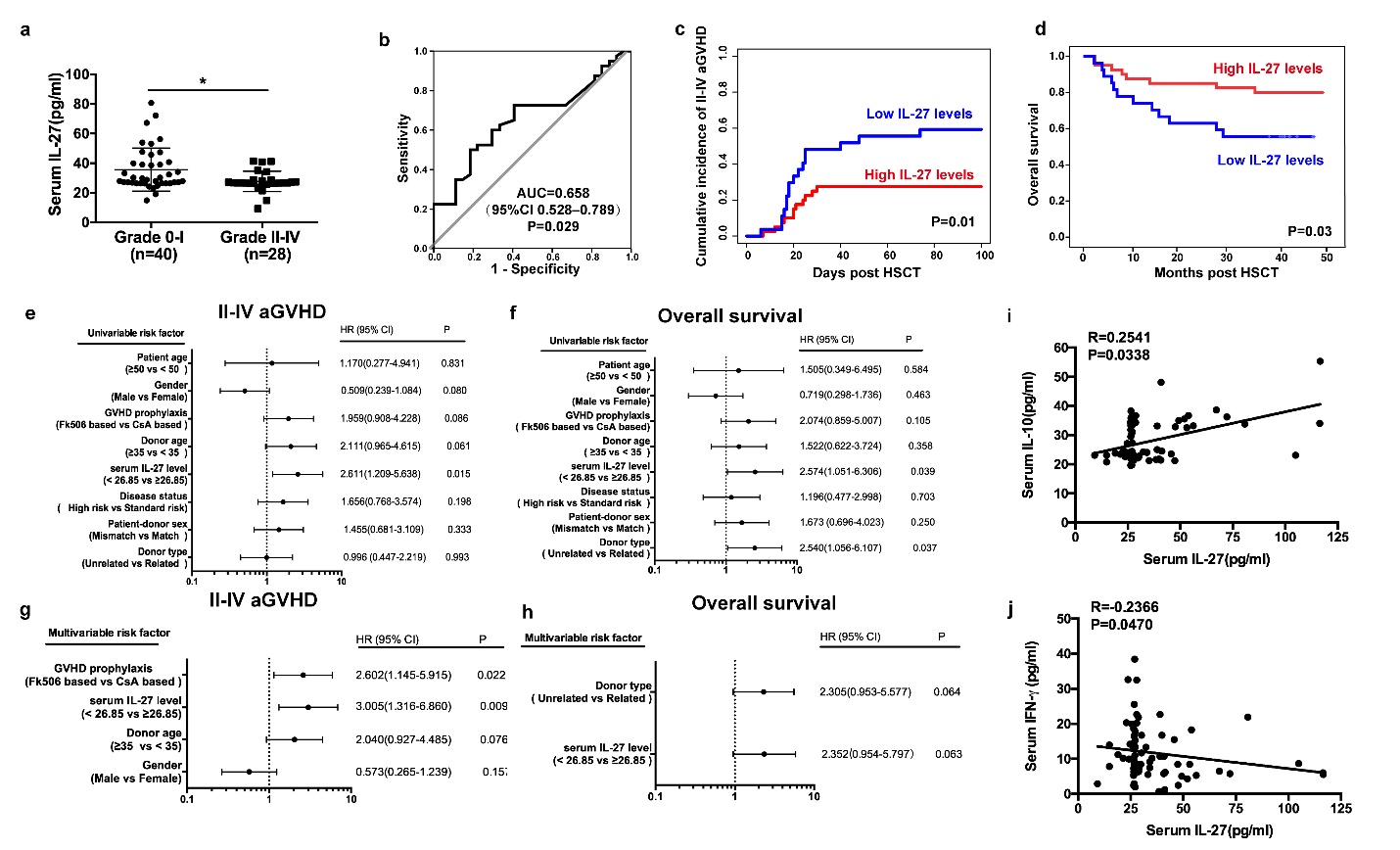


**Fig. S10 IL-27 is a valuable marker for predicting aGVHD after allo-HSCT in humans. a** IL-27 levels in patients with severe aGVHD (grade II-IV, n=28) and no/low-grade aGVHD (n=40) were examined by ELISA. **b** ROC curve was constructed to predict the occurrence of acute GVHD and the AUC was 0.658 (95% CI 0.528–0.78; P = 0.029). **c, d** The cumulative incidence of severe aGVHD **(c)** and overall survival **(d)** between patients with high and low IL-27 levels are shown. **e, f** Univariate analyses of factors affecting the incidence of severe aGVHD **(e)** or overall survival **(f)** after all-HSCT. **g, h** Multivariate analyses of factors affecting the incidence of severe aGVHD **(g)** or overall survival **(h)** after allo-HSCT. **i, j** The associations between serum levels of IL-27 and IL-10 **(i)**, and IFN-γ **(j)** were analyzed. Data are presented as mean ± SD. *, P < 0.05.

**Fig. S11.**


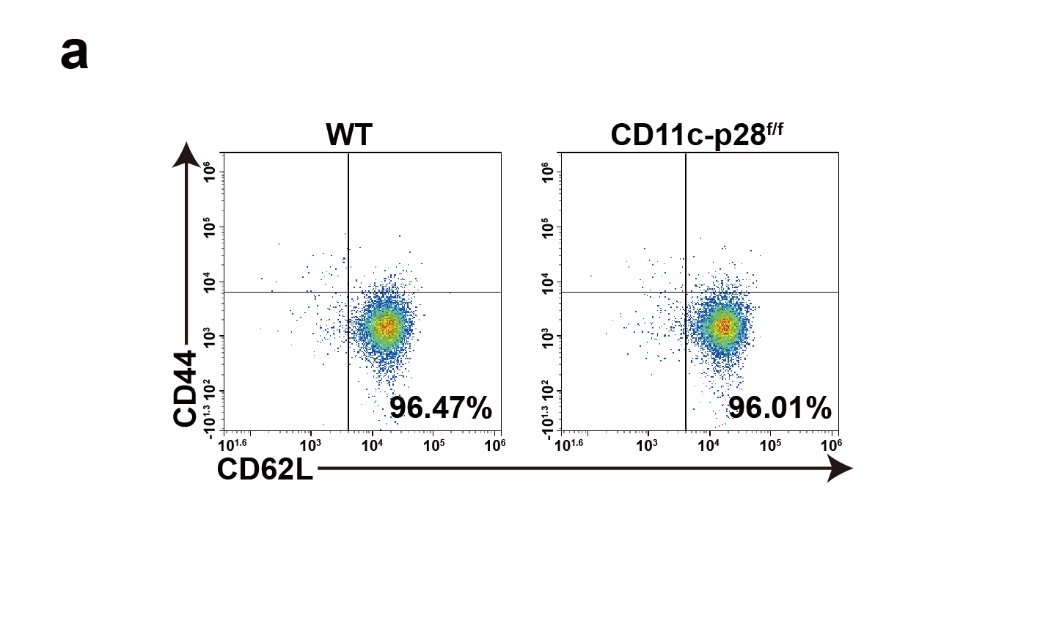


**Fig. S11 The purity of naïve T cells before transplantation. a** Naïve T cells were sorted from spleens of WT and CD11c-p28^f/f^ donors and the purity was determined by flow cytometry.**Table S1.** **The association of IL-27 p28 levels at pre-conditioning with clinical factors**

| Factors | Total | IL-27p28≥31.82 | IL-27<31.82 | *P* value |
| --- | --- | --- | --- | --- |
| Age median | 28(3-59) | 30(3-53) | 28(14-59) | 0.955 |
| Gender |  |  |  |  |
| male | 39 | 28 | 11 | 0.326 |
| female | 28 | 13 | 15 |  |
| Donor age | 34(16-55) | 34(26-50) | 46(20-55) | 0.322 |
| Patient-donor  sex match |  |  |  |  |
| match | 41 | 24 | 17 | 0.226 |
| mismatch | 26 | 17 | 9 |  |
| GVHD prophylaxis |  |  |  |  |
| CsA based | 47 | 29 | 18 | 0.22 |
| Fk506 based | 20 | 12 | 8 |  |
| Donor type |  |  |  |  |
| related | 46 | 29 | 17 | 0.535 |
| unrelated | 21 | 12 | 9 |  |
| Diagnosis |  |  |  |  |
| AML | 27 | 18 | 11 | 0.309 |
| ALL | 23 | 10 | 11 |  |
| MDS | 6 | 6 | 0 |  |
| CML | 11 | 7 | 4 |  |
| Disease status |  |  |  |  |
| standard risk | 48 | 31 | 17 | 0.065 |
| high risk | 19 | 10 | 9 |  |
| aGVHD grade |  |  |  |  |
| 0 | 14 | 13 | 1 | 0.003 |
| I | 26 | 18 | 8 |  |
| II | 18 | 9 | 11 |  |
| III | 4 | 0 | 4 |  |
| IV | 5 | 1 | 2 |  |
| II-IV aGVHD ggvgvaGVHD organ |  |  |  |  |
| skin | 18 | 6 | 12 | 0.004 |
| colon | 16 | 4 | 12 |  |
| liver | 5 | 1 | 4 |  |
| Prognosis |  |  |  |  |
| survival | 51 | 32 | 19 | 0.366 |
| relapse | 8 | 4 | 4 |  |
| other | 8 | 5 | 3 |  |

**Table S2.** **The association of sIL-27 levels at pre-conditioning with clinical factors**

| Factors | Total | IL-27≥26.85 | IL-27<26.85 | *P* value |
| --- | --- | --- | --- | --- |
| Age median | 28(3-59) | 32(3-59) | 25(12-52) | 0.074 |
| Gender |  |  |  |  |
| male | 41 | 28 | 13 | 0.072 |
| female | 26 | 12 | 14 |  |
| Donor age | 34(18-55) | 34(20-55) | 36(18-52) | 0.995 |
| Patient-donor  sex match |  |  |  |  |
| match | 41 | 26 | 15 | 0.436 |
| mismatch | 26 | 14 | 12 |  |
| GVHD prophylaxis |  |  |  |  |
| CsA based | 47 | 26 | 21 | 0.262 |
| Fk506 based | 20 | 14 | 6 |  |
| Donor type |  |  |  |  |
| related | 46 | 29 | 17 | 0.409 |
| unrelated | 21 | 11 | 10 |  |
| Diagnosis |  |  |  |  |
| AML | 27 | 18 | 9 | 0.309 |
| ALL | 23 | 12 | 11 |  |
| MDS | 6 | 2 | 4 |  |
| CML | 11 | 8 | 3 |  |
| Disease status |  |  |  |  |
| standard risk | 45 | 24 | 21 | 0.129 |
| high risk | 22 | 16 | 6 |  |
| aGVHD grade |  |  |  |  |
| 0 | 14 | 8 | 6 | 0.029 |
| I | 26 | 21 | 5 |  |
| II | 18 | 9 | 9 |  |
| III | 5 | 1 | 4 |  |
| IV | 4 | 1 | 3 |  |
| II-IV aGVHD ggvgvaGVHD organ |  |  |  |  |
| skin | 16 | 5 | 11 | 0.731 |
| colon | 10 | 4 | 6 |  |
| liver | 5 | 1 | 4 |  |
| Prognosis |  |  |  |  |
| survival | 47 | 32 | 15 | 0.034 |
| relapse | 9 | 2 | 7 |  |
| other | 11 | 6 | 5 |  |

Table S3. Differential gene expressions between Cluster 0 and Cluster 1

|  | **p_val** | **avg_log2FC(Cluster1 vs. 0)** | **pct.1(Cluster1)** | **pct.2(Cluster0)** | **p_val_adj** |
| --- | --- | --- | --- | --- | --- |
| **Il1r2** | 8.14E-43 | 2.261699272 | 0.502 | 0.046 | 1.71E-38 |
| **Tigit** | 6.04E-32 | 1.723556746 | 0.604 | 0.155 | 1.27E-27 |
| **Art2b** | 2.03E-30 | 2.018077947 | 0.367 | 0.032 | 4.27E-26 |
| **Srgn** | 8.64E-29 | 1.339168972 | 0.882 | 0.667 | 1.81E-24 |
| **Pdcd1** | 1.32E-27 | 1.650748205 | 0.527 | 0.141 | 2.76E-23 |
| **Lag3** | 2.05E-27 | 1.733587622 | 0.433 | 0.078 | 4.31E-23 |
| **Batf** | 5.68E-26 | 1.686957309 | 0.588 | 0.214 | 1.19E-21 |
| **Sdf4** | 3.65E-24 | 1.55378871 | 0.731 | 0.379 | 7.67E-20 |
| **Rps11** | 1.48E-23 | -0.602054549 | 0.976 | 0.983 | 3.11E-19 |
| **Cd74** | 1.79E-22 | 1.642158561 | 0.596 | 0.243 | 3.75E-18 |
| **Eea1** | 5.24E-22 | 1.547846756 | 0.392 | 0.083 | 1.1E-17 |
| **Sostdc1** | 1.2E-21 | 2.394888011 | 0.298 | 0.036 | 2.52E-17 |
| **Art2a-ps** | 5.45E-21 | 1.584855781 | 0.273 | 0.027 | 1.14E-16 |
| **Gm30211** | 8.57E-18 | 1.695199565 | 0.229 | 0.022 | 1.8E-13 |
| **Tnfrsf9** | 9.35E-18 | 1.711018669 | 0.359 | 0.092 | 1.96E-13 |
| **Rps24** | 1.24E-17 | -0.436730735 | 1 | 0.995 | 2.61E-13 |
| **Igkc** | 8.05E-17 | 1.618325923 | 0.278 | 0.049 | 1.69E-12 |
| **Foxp3** | 1.06E-16 | 1.262506989 | 0.229 | 0.027 | 2.23E-12 |
| **Dap** | 1.95E-16 | 1.394789577 | 0.437 | 0.16 | 4.08E-12 |
| **Thy1** | 1.18E-15 | -1.43665654 | 0.184 | 0.493 | 2.48E-11 |
| **Rgs16** | 2.21E-15 | 1.323604358 | 0.273 | 0.056 | 4.63E-11 |
| **Bhlhe40** | 2.25E-15 | 1.149520542 | 0.367 | 0.107 | 4.73E-11 |
| **Stx11** | 2.35E-15 | 1.187898938 | 0.253 | 0.044 | 4.92E-11 |
| **Cd83** | 3.08E-15 | 1.373646014 | 0.371 | 0.114 | 6.45E-11 |
| **Rps5** | 7.6E-15 | -0.436498663 | 0.98 | 0.985 | 1.59E-10 |
| **Rps16** | 1.66E-14 | -0.433486915 | 0.971 | 0.985 | 3.48E-10 |
| **Ifi27l2a** | 3.51E-14 | 0.761154454 | 0.976 | 0.859 | 7.36E-10 |
| **Tpi1** | 4.71E-14 | 1.198912111 | 0.384 | 0.138 | 9.89E-10 |
| **Mrps6** | 8.72E-14 | 1.017708361 | 0.359 | 0.117 | 1.83E-09 |
| **Aldoa** | 7.88E-13 | 0.973386405 | 0.649 | 0.454 | 1.65E-08 |
| **Gimap5** | 1.06E-12 | 1.080804428 | 0.408 | 0.165 | 2.23E-08 |
| **Pglyrp1** | 2.34E-12 | 1.06705953 | 0.4 | 0.16 | 4.91E-08 |
| **Rps13** | 2.58E-12 | -0.404774503 | 0.976 | 0.985 | 5.42E-08 |
| **Tbc1d4** | 2.89E-12 | 1.130197634 | 0.408 | 0.167 | 6.05E-08 |
| **Cd81** | 3.29E-12 | 1.196261895 | 0.363 | 0.133 | 6.9E-08 |
| **Rpl18a** | 8.4E-12 | -0.382988203 | 0.98 | 0.988 | 1.76E-07 |
| **Ftl1** | 1.04E-11 | 0.573575919 | 0.935 | 0.85 | 2.18E-07 |
| **Cd3e** | 1.74E-11 | 0.590867055 | 0.906 | 0.769 | 3.65E-07 |
| **Rps4x** | 3.08E-11 | -0.355188689 | 0.984 | 0.995 | 6.47E-07 |
| **Hif1a** | 3.89E-11 | 1.059933273 | 0.457 | 0.233 | 8.17E-07 |
| **Tnfrsf4** | 7.08E-11 | 0.860354694 | 0.592 | 0.328 | 1.49E-06 |
| **Cyfip1** | 1.89E-10 | 0.935341383 | 0.229 | 0.058 | 3.96E-06 |
| **Rps21** | 2.17E-10 | -0.370930055 | 0.976 | 0.981 | 4.54E-06 |
| **Cd82** | 2.26E-10 | 0.730911866 | 0.633 | 0.379 | 4.75E-06 |
| **Rilpl2** | 3.05E-10 | 1.048011812 | 0.384 | 0.175 | 6.41E-06 |
| **Sccpdh** | 3.33E-10 | 1.04868033 | 0.139 | 0.017 | 6.98E-06 |
| **Ascl2** | 3.41E-10 | 1.115779438 | 0.122 | 0.01 | 7.15E-06 |
| **Rpl36** | 5.57E-10 | -0.376185243 | 0.951 | 0.988 | 1.17E-05 |
| **Ptpn18** | 8.92E-10 | -0.642249658 | 0.604 | 0.769 | 1.87E-05 |
| **Slc26a2** | 9.65E-10 | 0.917050322 | 0.176 | 0.036 | 2.02E-05 |
| **Cst7** | 1.53E-09 | 1.113778652 | 0.461 | 0.245 | 3.21E-05 |
| **Ptpn1** | 1.62E-09 | 0.749704545 | 0.433 | 0.216 | 3.39E-05 |
| **Mif** | 1.97E-09 | 0.849391063 | 0.62 | 0.413 | 4.13E-05 |
| **Gapdh** | 2.4E-09 | 0.710969289 | 0.755 | 0.583 | 5.04E-05 |
| **Rpl23** | 2.52E-09 | -0.311194233 | 0.984 | 0.985 | 5.3E-05 |
| **Rps27** | 2.62E-09 | -0.350183925 | 0.992 | 0.993 | 5.5E-05 |
| **Rpl27a** | 2.68E-09 | -0.354314281 | 0.976 | 0.988 | 5.63E-05 |
| **Ctsb** | 2.75E-09 | 0.813461164 | 0.445 | 0.233 | 5.78E-05 |
| **Rpl17** | 3.38E-09 | -0.434865047 | 0.882 | 0.927 | 7.1E-05 |
| **Ighm** | 3.51E-09 | 0.569781683 | 0.629 | 0.391 | 7.38E-05 |
| **7-Sep** | 5.43E-09 | 0.812845348 | 0.359 | 0.16 | 0.000113926 |
| **Rpl8** | 7E-09 | -0.337049352 | 0.967 | 0.968 | 0.000146957 |
| **Rps20** | 7.56E-09 | -0.362468055 | 0.984 | 0.995 | 0.000158625 |
| **Izumo1r** | 8.76E-09 | 0.710538249 | 0.694 | 0.471 | 0.000183741 |
| **Itm2c** | 9.93E-09 | 0.846257241 | 0.49 | 0.279 | 0.000208427 |
| **Gdpd5** | 1.08E-08 | 0.684369593 | 0.106 | 0.01 | 0.000225722 |
| **Ppia** | 2.17E-08 | 0.32529079 | 0.992 | 0.964 | 0.00045492 |
| **Hsp90ab1** | 2.61E-08 | 0.475596916 | 0.89 | 0.801 | 0.000548045 |
| **Selplg** | 3.14E-08 | -1.101865196 | 0.155 | 0.342 | 0.000659237 |
| **Rpl19** | 3.58E-08 | -0.266303022 | 0.984 | 0.995 | 0.000752054 |
| **Bcl2a1b** | 3.69E-08 | 0.76194738 | 0.343 | 0.158 | 0.000773809 |
| **Rpl9** | 3.71E-08 | -0.283573911 | 0.992 | 0.993 | 0.000778843 |
| **2310001H17Rik** | 4.15E-08 | 0.838923548 | 0.371 | 0.187 | 0.000871506 |
| **Fam162a** | 4.98E-08 | 0.875727013 | 0.335 | 0.155 | 0.001044801 |
| **Rps3** | 5.27E-08 | -0.331682322 | 0.955 | 0.981 | 0.001106512 |
| **Tox2** | 5.57E-08 | 0.538843191 | 0.151 | 0.032 | 0.001169116 |
| **Ikzf2** | 7.06E-08 | 0.698404773 | 0.539 | 0.316 | 0.001480552 |
| **Cd2** | 9.87E-08 | 0.5589518 | 0.678 | 0.439 | 0.002071797 |
| **Vamp5** | 1.07E-07 | 0.764250729 | 0.229 | 0.08 | 0.002255238 |
| **Flnb** | 1.19E-07 | 0.82165277 | 0.143 | 0.032 | 0.002495148 |
| **Cxcr6** | 1.64E-07 | -0.999921022 | 0.012 | 0.131 | 0.003434019 |
| **Grcc10** | 1.69E-07 | 0.590839057 | 0.637 | 0.449 | 0.003540208 |
| **Rpl10a** | 1.86E-07 | -0.407689612 | 0.886 | 0.91 | 0.003893048 |
| **Tshz2** | 1.91E-07 | 0.61625521 | 0.11 | 0.017 | 0.004002259 |
| **Rps28** | 1.98E-07 | -0.339886252 | 0.947 | 0.966 | 0.004155712 |
| **S100a11** | 2.11E-07 | 0.533751689 | 0.637 | 0.434 | 0.004428279 |
| **Casp4** | 3.07E-07 | 0.719590072 | 0.151 | 0.039 | 0.006437974 |
| **Ndfip1** | 3.19E-07 | 0.659783441 | 0.616 | 0.442 | 0.00668791 |
| **Rps23** | 3.5E-07 | -0.327471377 | 0.947 | 0.954 | 0.007342043 |
| **Malt1** | 3.58E-07 | 0.797595549 | 0.237 | 0.09 | 0.007518703 |
| **Rplp1** | 3.69E-07 | -0.307051607 | 0.976 | 0.983 | 0.007732998 |
| **Zc3h12d** | 3.76E-07 | 0.684077195 | 0.155 | 0.041 | 0.007899735 |
| **Rpl30** | 4.1E-07 | -0.25328449 | 0.992 | 0.993 | 0.008609619 |
| **Tnfrsf1b** | 4.56E-07 | 0.845138592 | 0.208 | 0.075 | 0.009573328 |
| **Tnfsf8** | 4.7E-07 | 0.873476416 | 0.29 | 0.131 | 0.009860167 |
| **Twsg1** | 6.22E-07 | 0.777634487 | 0.147 | 0.039 | 0.013043136 |
| **H2-K1** | 6.25E-07 | 0.358128551 | 0.988 | 0.927 | 0.013120396 |
| **Hmgn3** | 6.57E-07 | 0.707551524 | 0.188 | 0.063 | 0.013796588 |
| **Rps15a** | 7.19E-07 | -0.254838278 | 0.992 | 0.995 | 0.01508803 |
| **Rpl12** | 7.22E-07 | -0.451673203 | 0.767 | 0.828 | 0.015148095 |
| **Rps14** | 7.62E-07 | -0.315272863 | 0.91 | 0.942 | 0.015987246 |
| **Ptms** | 7.82E-07 | 0.724709385 | 0.282 | 0.129 | 0.016405533 |
| **Rab11a** | 7.88E-07 | 0.759719149 | 0.253 | 0.104 | 0.016540324 |
| **Satb1** | 8.14E-07 | -1.074881641 | 0.09 | 0.24 | 0.017078068 |
| **Lmo4** | 1.01E-06 | -1.199442768 | 0.041 | 0.167 | 0.021210915 |
| **Znrf1** | 1.03E-06 | -0.910211463 | 0.053 | 0.187 | 0.021666308 |
| **Chd9** | 1.05E-06 | 0.759090991 | 0.257 | 0.114 | 0.022070846 |
| **H2afz** | 1.05E-06 | 0.57830749 | 0.767 | 0.675 | 0.022136291 |
| **Slc7a10** | 1.12E-06 | 0.539541827 | 0.102 | 0.017 | 0.023540126 |
| **Rpl32** | 1.36E-06 | -0.310672318 | 0.971 | 0.985 | 0.028598997 |
| **Axl** | 1.4E-06 | 0.687040307 | 0.147 | 0.041 | 0.029327939 |
| **Pdcd1lg2** | 1.41E-06 | 0.751610973 | 0.171 | 0.056 | 0.029557022 |
| **Angptl2** | 1.47E-06 | 0.593794759 | 0.106 | 0.019 | 0.030878405 |
| **Tiam1** | 1.48E-06 | 0.729537754 | 0.273 | 0.126 | 0.031095611 |
| **Relb** | 1.6E-06 | 0.710927951 | 0.155 | 0.046 | 0.033606574 |
| **Gstp3** | 1.72E-06 | 0.709288812 | 0.302 | 0.146 | 0.036067265 |
| **Gm42031** | 1.82E-06 | 0.938398573 | 0.2 | 0.078 | 0.038125117 |
| **Cd53** | 1.9E-06 | 0.539825097 | 0.49 | 0.301 | 0.039797305 |
| **Kbtbd11** | 1.96E-06 | -0.752153421 | 0.024 | 0.138 | 0.041090917 |
| **Txnip** | 2.17E-06 | -0.899483912 | 0.098 | 0.245 | 0.045606664 |
| **Psme2** | 2.52E-06 | 0.634631618 | 0.624 | 0.464 | 0.052812416 |
| **Rps9** | 2.52E-06 | -0.288809669 | 0.959 | 0.985 | 0.052834497 |
| **Gdpd3** | 2.73E-06 | 0.584506672 | 0.514 | 0.33 | 0.057204539 |
| **Rpl18** | 2.81E-06 | -0.263730032 | 0.971 | 0.985 | 0.058934138 |
| **Serinc3** | 3.01E-06 | 0.575488469 | 0.453 | 0.274 | 0.063120211 |
| **Ms4a4b** | 3.51E-06 | -0.826833157 | 0.171 | 0.333 | 0.073577011 |
| **Klf2** | 3.7E-06 | -0.923503093 | 0.045 | 0.165 | 0.077601652 |
| **Il7r** | 3.89E-06 | -0.880406519 | 0.249 | 0.41 | 0.081550768 |
| **Dus2** | 4.02E-06 | 0.44542767 | 0.151 | 0.046 | 0.084451872 |
| **Eif1** | 4.43E-06 | 0.302018521 | 0.918 | 0.891 | 0.092887484 |
| **Rps12** | 5.53E-06 | -0.373341763 | 0.914 | 0.934 | 0.116004904 |
| **Cd8b1** | 5.62E-06 | -1.276178032 | 0.065 | 0.189 | 0.11784039 |
| **Cdk6** | 5.63E-06 | 0.706436791 | 0.127 | 0.034 | 0.118223486 |
| **Rps18** | 6.13E-06 | -0.336528801 | 0.914 | 0.949 | 0.128597614 |
| **Serp1** | 6.48E-06 | 0.605301707 | 0.318 | 0.167 | 0.135976316 |
| **Gpm6b** | 6.91E-06 | 0.437555136 | 0.151 | 0.046 | 0.144996172 |
| **Trac** | 7.46E-06 | 0.538291723 | 0.608 | 0.422 | 0.156477409 |
| **Atp1b1** | 8.44E-06 | 0.694002665 | 0.114 | 0.029 | 0.177016292 |
| **Tnfaip8** | 9.12E-06 | 0.635163155 | 0.355 | 0.201 | 0.191439493 |
| **Tspan3** | 1.01E-05 | 0.813595455 | 0.241 | 0.114 | 0.211877414 |
| **Itgav** | 1.14E-05 | 0.730638179 | 0.155 | 0.053 | 0.238390867 |
| **Nfkb2** | 1.18E-05 | 0.746213627 | 0.2 | 0.083 | 0.247386654 |
| **Hspa5** | 1.2E-05 | 0.437327875 | 0.437 | 0.26 | 0.251321767 |
| **Ifngr1** | 1.26E-05 | -1.166751187 | 0.114 | 0.243 | 0.264438232 |
| **Kdm2b** | 1.32E-05 | 0.543799717 | 0.163 | 0.058 | 0.277927661 |
| **Gadd45b** | 1.39E-05 | 0.514251933 | 0.318 | 0.163 | 0.291740238 |
| **Adora2a** | 1.4E-05 | 0.54852594 | 0.155 | 0.053 | 0.294724667 |
| **Ttn** | 1.52E-05 | 0.567584362 | 0.143 | 0.046 | 0.318708737 |
| **Gm43305** | 1.55E-05 | 0.779312035 | 0.163 | 0.061 | 0.325532814 |
| **Prdx1** | 1.6E-05 | 0.561339163 | 0.429 | 0.267 | 0.335431984 |
| **Pkp3** | 1.86E-05 | 0.558432156 | 0.376 | 0.216 | 0.389466647 |
| **Rnh1** | 1.94E-05 | 0.622864963 | 0.2 | 0.085 | 0.407983484 |
| **Icam1** | 2.24E-05 | 0.595833626 | 0.155 | 0.056 | 0.471077392 |
| **Ccdc28b** | 2.3E-05 | 0.631771982 | 0.184 | 0.075 | 0.481848106 |
| **Eif4ebp1** | 2.35E-05 | 0.549220986 | 0.135 | 0.044 | 0.493890565 |
| **Prnp** | 2.47E-05 | 0.431492227 | 0.102 | 0.024 | 0.518240557 |
| **Igf1r** | 2.61E-05 | 0.58421954 | 0.184 | 0.075 | 0.547262693 |
| **Cst3** | 2.69E-05 | 0.450387242 | 0.371 | 0.209 | 0.564233693 |
| **Ctsw** | 2.9E-05 | -0.712208845 | 0.016 | 0.102 | 0.608282808 |
| **B9d2** | 3.12E-05 | 0.594268371 | 0.155 | 0.058 | 0.65380336 |
| **Ccr7** | 4E-05 | -0.763078933 | 0.029 | 0.121 | 0.838529848 |
| **Cox6c** | 4.02E-05 | 0.37832239 | 0.755 | 0.602 | 0.844039021 |
| **Grap2** | 4.45E-05 | -0.698092536 | 0.029 | 0.119 | 0.934466356 |
| **AU020206** | 4.51E-05 | 0.539927586 | 0.273 | 0.143 | 0.947417757 |
| **Rpl36a** | 4.6E-05 | -0.354062645 | 0.845 | 0.879 | 0.964588535 |
| **Sqstm1** | 4.87E-05 | 0.514135643 | 0.371 | 0.226 | 1 |
| **Smco4** | 4.92E-05 | 0.562335122 | 0.351 | 0.206 | 1 |
| **Cstb** | 5.22E-05 | 0.637831252 | 0.245 | 0.126 | 1 |
| **Anxa6** | 5.66E-05 | -0.62976199 | 0.061 | 0.172 | 1 |
| **Saraf** | 5.88E-05 | -0.718481514 | 0.163 | 0.296 | 1 |
| **Pfn1** | 6.32E-05 | 0.252918855 | 0.976 | 0.966 | 1 |
| **Pkm** | 6.53E-05 | 0.520058501 | 0.514 | 0.379 | 1 |
| **Rps26** | 6.83E-05 | -0.272426707 | 0.951 | 0.964 | 1 |
| **Ctsz** | 6.96E-05 | 0.541093175 | 0.212 | 0.1 | 1 |
| **Rnf19a** | 7.34E-05 | 0.569965481 | 0.151 | 0.058 | 1 |
| **Cdc25b** | 7.42E-05 | 0.37203751 | 0.143 | 0.051 | 1 |
| **Cd8a** | 7.45E-05 | -0.810233137 | 0.045 | 0.143 | 1 |
| **Prdx6** | 7.71E-05 | -0.715533216 | 0.151 | 0.277 | 1 |
| **Rpl35** | 7.71E-05 | -0.259671199 | 0.951 | 0.959 | 1 |
| **Pnkd** | 7.98E-05 | 0.542493292 | 0.233 | 0.117 | 1 |
| **Egr2** | 8E-05 | 0.623049266 | 0.102 | 0.029 | 1 |
| **S100a4** | 8.03E-05 | -0.866714372 | 0.086 | 0.199 | 1 |
| **Hcfc1** | 8.56E-05 | 0.513458821 | 0.118 | 0.039 | 1 |
| **Ptpn11** | 9.46E-05 | 0.778968315 | 0.229 | 0.119 | 1 |
| **Fth1** | 9.97E-05 | 0.270914508 | 0.967 | 0.947 | 1 |
| **Gem** | 0.000100146 | 0.522439847 | 0.102 | 0.029 | 1 |
| **Nfkbia** | 0.000108051 | 0.618859559 | 0.645 | 0.536 | 1 |
| **Rpl27** | 0.000109182 | -0.323590422 | 0.869 | 0.871 | 1 |
| **Tank** | 0.000109816 | 0.431081457 | 0.204 | 0.092 | 1 |
| **Ptp4a2** | 0.000110884 | 0.590126299 | 0.335 | 0.209 | 1 |
| **Ckb** | 0.000112342 | -0.810756897 | 0.02 | 0.1 | 1 |
| **Pou2f2** | 0.000114046 | 0.46812363 | 0.29 | 0.158 | 1 |
| **Gna13** | 0.000126898 | 0.712484964 | 0.224 | 0.117 | 1 |
| **Ptma** | 0.000128737 | 0.374791528 | 0.878 | 0.818 | 1 |
| **Nrn1** | 0.000130217 | 0.342199672 | 0.229 | 0.109 | 1 |
| **Pgk1** | 0.000132469 | 0.682802068 | 0.29 | 0.172 | 1 |
| **Rpl26** | 0.000135918 | -0.258817012 | 0.902 | 0.913 | 1 |
| **Traf1** | 0.000136755 | 0.599019573 | 0.31 | 0.182 | 1 |
| **Marcksl1** | 0.000137894 | 0.694680428 | 0.212 | 0.107 | 1 |
| **Mien1** | 0.000143645 | -0.5985471 | 0.131 | 0.255 | 1 |
| **Cd3d** | 0.000157225 | 0.382627728 | 0.849 | 0.731 | 1 |
| **Glipr2** | 0.000172613 | -0.566967112 | 0.024 | 0.104 | 1 |
| **Eif4enif1** | 0.000177012 | 0.492496189 | 0.11 | 0.036 | 1 |
| **Btla** | 0.000179864 | 0.503335145 | 0.11 | 0.036 | 1 |
| **Nfil3** | 0.000181889 | 0.564146363 | 0.102 | 0.032 | 1 |
| **Lat** | 0.000197189 | -0.459312633 | 0.551 | 0.648 | 1 |
| **Uba52** | 0.000197325 | -0.455917696 | 0.747 | 0.779 | 1 |
| **Ddx24** | 0.000204641 | 0.429370755 | 0.363 | 0.233 | 1 |
| **Vim** | 0.000223382 | -0.671874994 | 0.286 | 0.417 | 1 |
| **Rpl14** | 0.000231289 | -0.363173881 | 0.829 | 0.852 | 1 |
| **Max** | 0.000237448 | 0.45190695 | 0.257 | 0.141 | 1 |
| **Sit1** | 0.00024301 | -0.52168329 | 0.053 | 0.148 | 1 |
| **Esyt1** | 0.00028124 | -0.56657594 | 0.057 | 0.15 | 1 |
| **Sar1b** | 0.000317608 | 0.50243582 | 0.167 | 0.078 | 1 |
| **Ahi1** | 0.000326656 | 0.544103808 | 0.102 | 0.034 | 1 |
| **Mmd** | 0.000334947 | 0.66159515 | 0.167 | 0.08 | 1 |
| **Raf1** | 0.000372178 | 0.409833708 | 0.167 | 0.075 | 1 |
| **Sigirr** | 0.000379463 | 0.724231157 | 0.163 | 0.078 | 1 |
| **Glipr1** | 0.000389026 | 0.513106837 | 0.143 | 0.061 | 1 |
| **Tnfrsf18** | 0.000414272 | 0.601262826 | 0.449 | 0.316 | 1 |
| **Cdk2ap2** | 0.000418006 | 0.38671789 | 0.657 | 0.529 | 1 |
| **Acp5** | 0.00042957 | -0.615291995 | 0.069 | 0.165 | 1 |
| **Klf6** | 0.000441056 | -0.722332074 | 0.078 | 0.172 | 1 |
| **Rbl2** | 0.000461354 | -0.60235139 | 0.057 | 0.146 | 1 |
| **Arid5b** | 0.000470586 | 0.442928135 | 0.171 | 0.08 | 1 |
| **Mrps25** | 0.000476966 | 0.515051841 | 0.118 | 0.046 | 1 |
| **Cdkn2d** | 0.000504922 | -0.593885611 | 0.069 | 0.163 | 1 |
| **Prelid1** | 0.000510867 | 0.413721242 | 0.551 | 0.422 | 1 |
| **Pim1** | 0.000541566 | 0.563075691 | 0.253 | 0.146 | 1 |
| **Tmem256** | 0.000550858 | 0.364524805 | 0.31 | 0.184 | 1 |
| **Il18r1** | 0.000554711 | -0.735382383 | 0.041 | 0.119 | 1 |
| **Gm17745** | 0.000567599 | 0.485347895 | 0.102 | 0.036 | 1 |
| **Hdac7** | 0.0005696 | 0.610443995 | 0.22 | 0.121 | 1 |
| **Gm20400** | 0.000582156 | 0.78517425 | 0.131 | 0.056 | 1 |
| **Ergic1** | 0.000652183 | 0.478121113 | 0.147 | 0.066 | 1 |
| **Dusp1** | 0.000653625 | 0.256637775 | 0.502 | 0.362 | 1 |
| **Penk** | 0.000660185 | 1.121118533 | 0.147 | 0.066 | 1 |
| **Ccr8** | 0.000660977 | 0.519164603 | 0.139 | 0.061 | 1 |
| **Lrrfip2** | 0.000680008 | 0.354238623 | 0.11 | 0.041 | 1 |
| **Asb2** | 0.000716436 | 0.545697899 | 0.159 | 0.075 | 1 |
| **Lamp1** | 0.000723403 | 0.525081161 | 0.208 | 0.112 | 1 |
| **Ankrd44** | 0.000726998 | -0.54971246 | 0.082 | 0.175 | 1 |
| **Dgkd** | 0.00072938 | -0.546267924 | 0.037 | 0.112 | 1 |
| **Ubash3b** | 0.000741068 | 0.528905329 | 0.118 | 0.049 | 1 |
| **Map4k2** | 0.000741668 | -0.448137647 | 0.057 | 0.146 | 1 |
| **Zap70** | 0.000779231 | 0.486639497 | 0.38 | 0.262 | 1 |
| **Mapk1** | 0.000797173 | 0.585317431 | 0.167 | 0.083 | 1 |
| **Cox17** | 0.000816939 | 0.393750039 | 0.367 | 0.252 | 1 |
| **Rcsd1** | 0.000831153 | -0.629465681 | 0.094 | 0.187 | 1 |
| **Tox** | 0.000840755 | 0.558315213 | 0.29 | 0.184 | 1 |
| **Ost4** | 0.000882067 | 0.41642407 | 0.514 | 0.391 | 1 |
| **Mcrip1** | 0.000886584 | 0.385109213 | 0.143 | 0.066 | 1 |
| **Nab1** | 0.000888781 | 0.437452918 | 0.127 | 0.053 | 1 |
| **Cxcr4** | 0.000937633 | 0.282640598 | 0.204 | 0.104 | 1 |
| **Uqcr11** | 0.000941733 | 0.358196923 | 0.461 | 0.337 | 1 |
| **Ppp1r14b** | 0.000943539 | 0.892592325 | 0.196 | 0.112 | 1 |
| **Rpl28** | 0.001014674 | -0.27107377 | 0.91 | 0.903 | 1 |
| **Lfng** | 0.001032143 | -0.626582572 | 0.057 | 0.136 | 1 |
| **Hivep3** | 0.001053445 | 0.427836975 | 0.127 | 0.053 | 1 |
| **Gpx4** | 0.001069751 | 0.361665232 | 0.555 | 0.444 | 1 |
| **0610012G03Rik** | 0.001075238 | -0.525180198 | 0.069 | 0.155 | 1 |
| **Ncf4** | 0.001096329 | 0.395149825 | 0.171 | 0.085 | 1 |
| **Eef1d** | 0.001124021 | -0.377970285 | 0.437 | 0.575 | 1 |
| **Ldha** | 0.001138235 | 0.482490274 | 0.522 | 0.432 | 1 |
| **Ubb** | 0.001149701 | 0.326632104 | 0.902 | 0.801 | 1 |
| **Baz1a** | 0.001152322 | 0.332047703 | 0.122 | 0.051 | 1 |
| **Prelid2** | 0.001178582 | 0.440869068 | 0.122 | 0.051 | 1 |
| **Ccdc85b** | 0.001179814 | -0.503599396 | 0.086 | 0.18 | 1 |
| **Ahnak** | 0.001225739 | -0.5483198 | 0.147 | 0.252 | 1 |
| **Rps10** | 0.001236374 | -0.576458535 | 0.282 | 0.388 | 1 |
| **Stat4** | 0.0012581 | 0.457991157 | 0.135 | 0.061 | 1 |
| **Ptger2** | 0.001267561 | 0.480966958 | 0.155 | 0.075 | 1 |
| **Edf1** | 0.001281579 | 0.357717598 | 0.563 | 0.454 | 1 |
| **Rag1** | 0.001284255 | -0.83920105 | 0.033 | 0.1 | 1 |
| **Eloc** | 0.001284782 | 0.470349843 | 0.29 | 0.189 | 1 |
| **Fundc2** | 0.00129482 | 0.544749892 | 0.22 | 0.129 | 1 |
| **Cd28** | 0.001300994 | -0.539695899 | 0.212 | 0.328 | 1 |
| **Dck** | 0.001351028 | 0.398920274 | 0.131 | 0.058 | 1 |
| **Areg** | 0.001368206 | 0.317720119 | 0.114 | 0.046 | 1 |
| **Cnot8** | 0.001394713 | -0.450336089 | 0.037 | 0.107 | 1 |
| **Map2k3** | 0.001395497 | 0.483331266 | 0.163 | 0.083 | 1 |
| **Ms4a6b** | 0.001398837 | -0.56650252 | 0.229 | 0.335 | 1 |
| **Prkca** | 0.001436093 | 0.474136677 | 0.273 | 0.172 | 1 |
| **Uqcrq** | 0.001552961 | 0.367975147 | 0.469 | 0.34 | 1 |
| **M6pr** | 0.001576478 | 0.525812012 | 0.302 | 0.204 | 1 |
| **Id2** | 0.001596378 | -0.536265147 | 0.188 | 0.296 | 1 |
| **Klk8** | 0.001602588 | -0.460898647 | 0.106 | 0.204 | 1 |
| **Anapc11** | 0.001623788 | 0.404910989 | 0.347 | 0.231 | 1 |
| **B3gnt2** | 0.001634138 | 0.485182209 | 0.192 | 0.104 | 1 |
| **Hacd3** | 0.001646483 | 0.467269155 | 0.159 | 0.08 | 1 |
| **Med21** | 0.001708154 | 0.470175992 | 0.114 | 0.049 | 1 |
| **Ubl5** | 0.001726751 | 0.364291153 | 0.629 | 0.519 | 1 |
| **Swi5** | 0.001727835 | 0.440289785 | 0.363 | 0.252 | 1 |
| **Txn1** | 0.001731604 | 0.419975573 | 0.367 | 0.26 | 1 |
| **Camk4** | 0.001742044 | -0.465221644 | 0.065 | 0.148 | 1 |
| **Cyba** | 0.001763425 | 0.353397664 | 0.633 | 0.536 | 1 |
| **Nfkbiz** | 0.001801002 | 0.430073971 | 0.139 | 0.066 | 1 |
| **Gnb2** | 0.001850202 | 0.343514494 | 0.351 | 0.238 | 1 |
| **Lbr** | 0.001863481 | -0.464582289 | 0.065 | 0.146 | 1 |
| **Vta1** | 0.001958596 | 0.378779797 | 0.11 | 0.046 | 1 |
| **Cct4** | 0.002047885 | 0.411777719 | 0.249 | 0.153 | 1 |
| **Usp3** | 0.002090664 | -0.384426397 | 0.057 | 0.133 | 1 |
| **Wls** | 0.0020943 | 0.494658299 | 0.127 | 0.058 | 1 |
| **Psmd11** | 0.002102014 | 0.469847498 | 0.192 | 0.107 | 1 |
| **Slfn1** | 0.002115847 | -0.594707237 | 0.061 | 0.138 | 1 |
| **Phyh** | 0.002121216 | 0.337168592 | 0.114 | 0.049 | 1 |
| **Chmp4b** | 0.002133238 | 0.457720334 | 0.212 | 0.124 | 1 |
| **Puf60** | 0.00213418 | 0.434403025 | 0.265 | 0.165 | 1 |
| **Zc3h15** | 0.002138448 | 0.53429025 | 0.269 | 0.175 | 1 |
| **Smim4** | 0.002186049 | -0.384906429 | 0.045 | 0.117 | 1 |
| **Pfkl** | 0.00222012 | 0.331614669 | 0.106 | 0.044 | 1 |
| **Zfand5** | 0.002256743 | 0.363173161 | 0.106 | 0.044 | 1 |
| **Usp4** | 0.002328698 | 0.354206081 | 0.118 | 0.053 | 1 |
| **Resf1** | 0.002386475 | -0.610107473 | 0.102 | 0.187 | 1 |
| **Eif4a1** | 0.002414309 | 0.482111124 | 0.376 | 0.272 | 1 |
| **Casp3** | 0.00241559 | 0.391016725 | 0.135 | 0.063 | 1 |
| **Gm8995** | 0.002437622 | -0.645277158 | 0.086 | 0.167 | 1 |
| **Rtcb** | 0.002451291 | 0.303268663 | 0.163 | 0.085 | 1 |
| **Anxa2** | 0.002481568 | 0.528280135 | 0.216 | 0.129 | 1 |
| **Gpr132** | 0.002483014 | -0.441361829 | 0.045 | 0.114 | 1 |
| **Pdia6** | 0.002484801 | 0.537587143 | 0.167 | 0.09 | 1 |
| **Med15** | 0.002609869 | 0.551044332 | 0.171 | 0.095 | 1 |
| **Crip1** | 0.002653063 | -0.687523469 | 0.408 | 0.505 | 1 |
| **Vamp4** | 0.002681175 | -0.42540215 | 0.069 | 0.148 | 1 |
| **Mcm6** | 0.002700261 | 0.617337326 | 0.143 | 0.073 | 1 |
| **Arhgap45** | 0.002708977 | -0.301962721 | 0.535 | 0.65 | 1 |
| **Mdh1** | 0.002712384 | 0.418741139 | 0.302 | 0.199 | 1 |
| **Maf** | 0.00273037 | 0.426514396 | 0.384 | 0.279 | 1 |
| **Osbpl9** | 0.00278473 | 0.431858643 | 0.11 | 0.049 | 1 |
| **Ylpm1** | 0.002790795 | 0.532401724 | 0.106 | 0.046 | 1 |
| **Xist** | 0.002868759 | 0.321510948 | 0.612 | 0.507 | 1 |
| **Podnl1** | 0.002999391 | 0.378100263 | 0.118 | 0.053 | 1 |
| **Cish** | 0.003083279 | -0.593796504 | 0.073 | 0.15 | 1 |
| **Syt11** | 0.003084034 | 0.270385667 | 0.163 | 0.085 | 1 |
| **Ucp2** | 0.003111787 | 0.261399053 | 0.824 | 0.789 | 1 |
| **Ssr4** | 0.003147746 | 0.41370601 | 0.449 | 0.354 | 1 |
| **H3f3a** | 0.003152052 | 0.270789213 | 0.718 | 0.612 | 1 |
| **Itgb7** | 0.003190967 | -0.498337441 | 0.184 | 0.286 | 1 |
| **Chchd10** | 0.003195686 | 0.428370911 | 0.143 | 0.073 | 1 |
| **Shisa5** | 0.003275895 | 0.277219289 | 0.873 | 0.801 | 1 |
| **Arl3** | 0.003428383 | 0.358417303 | 0.102 | 0.044 | 1 |
| **Cflar** | 0.003452997 | 0.478826806 | 0.216 | 0.131 | 1 |
| **Grn** | 0.003541554 | 0.311284669 | 0.11 | 0.049 | 1 |
| **Odc1** | 0.00362151 | 0.418442466 | 0.2 | 0.117 | 1 |
| **Nfatc1** | 0.003659254 | 0.519238877 | 0.249 | 0.158 | 1 |
| **Fam102a** | 0.003730104 | -0.519621722 | 0.053 | 0.121 | 1 |
| **Fyn** | 0.003772932 | 0.468992929 | 0.298 | 0.204 | 1 |
| **Crlf3** | 0.003805205 | -0.55962274 | 0.09 | 0.167 | 1 |
| **Pebp1** | 0.003819799 | 0.438962705 | 0.367 | 0.272 | 1 |
| **Rrbp1** | 0.00383213 | 0.443242093 | 0.204 | 0.121 | 1 |
| **Ifi27** | 0.003855333 | 0.447255104 | 0.355 | 0.255 | 1 |
| **Nedd9** | 0.003895673 | 0.364045224 | 0.118 | 0.056 | 1 |
| **Cytip** | 0.003974889 | -0.668960424 | 0.208 | 0.296 | 1 |
| **Pip4k2a** | 0.004013311 | -0.436807715 | 0.041 | 0.104 | 1 |
| **Bsg** | 0.004024697 | 0.375940099 | 0.351 | 0.245 | 1 |
| **Socs1** | 0.004055574 | -0.647388132 | 0.249 | 0.337 | 1 |
| **Arap2** | 0.004155764 | 0.273294462 | 0.135 | 0.066 | 1 |
| **Rps6** | 0.004207981 | -0.290649226 | 0.771 | 0.782 | 1 |
| **Tgfb1** | 0.004242484 | 0.401224013 | 0.188 | 0.109 | 1 |
| **Ssbp1** | 0.004246301 | 0.429654372 | 0.188 | 0.112 | 1 |
| **Egr1** | 0.004280221 | 0.356735321 | 0.167 | 0.092 | 1 |
| **Fli1** | 0.004331229 | -0.481075443 | 0.061 | 0.131 | 1 |
| **Tmem154** | 0.00435178 | 0.366540844 | 0.167 | 0.092 | 1 |
| **Krcc1** | 0.004639996 | 0.438108881 | 0.237 | 0.15 | 1 |
| **Tgif1** | 0.004671987 | 0.303952797 | 0.127 | 0.061 | 1 |
| **Timp2** | 0.004817062 | 0.549774589 | 0.167 | 0.097 | 1 |
| **Polr2a** | 0.004824601 | 0.426654848 | 0.31 | 0.214 | 1 |
| **Rasgrp1** | 0.004851646 | -0.617573194 | 0.073 | 0.146 | 1 |
| **Pik3r1** | 0.004907935 | -0.428216333 | 0.049 | 0.114 | 1 |
| **Bcor** | 0.005043581 | 0.512600661 | 0.163 | 0.092 | 1 |
| **Rpp21** | 0.005409925 | -0.63651206 | 0.118 | 0.197 | 1 |
| **Ccr9** | 0.005486853 | -0.436342846 | 0.045 | 0.107 | 1 |
| **Psma5** | 0.005540312 | 0.338591818 | 0.237 | 0.148 | 1 |
| **Arhgef1** | 0.005786351 | -0.423529041 | 0.278 | 0.379 | 1 |
| **Ppp1r10** | 0.005811288 | 0.373848033 | 0.147 | 0.078 | 1 |
| **Sdhb** | 0.005849852 | 0.350439906 | 0.322 | 0.223 | 1 |
| **Nek7** | 0.005889427 | 0.338716766 | 0.131 | 0.066 | 1 |
| **Ube2b** | 0.00600472 | 0.405479442 | 0.327 | 0.233 | 1 |
| **Chchd2** | 0.006037119 | 0.256336495 | 0.645 | 0.519 | 1 |
| **Zfp207** | 0.006113842 | 0.444896875 | 0.167 | 0.097 | 1 |
| **Gimap8** | 0.006146271 | 0.329679019 | 0.163 | 0.09 | 1 |
| **Cd40lg** | 0.006179457 | -0.501761746 | 0.086 | 0.16 | 1 |
| **Pglyrp2** | 0.00621213 | -0.48537753 | 0.069 | 0.141 | 1 |
| **Tmed10** | 0.006347772 | 0.347502329 | 0.216 | 0.133 | 1 |
| **Uqcc2** | 0.006468919 | 0.499057786 | 0.237 | 0.16 | 1 |
| **Dapp1** | 0.006486824 | 0.391150412 | 0.122 | 0.061 | 1 |
| **Ubald2** | 0.006528926 | 0.517637395 | 0.278 | 0.194 | 1 |
| **Dynll1** | 0.006643583 | 0.433678324 | 0.478 | 0.376 | 1 |
| **Ehd1** | 0.006966378 | 0.301081402 | 0.118 | 0.058 | 1 |
| **Slc44a2** | 0.007087292 | -0.440051674 | 0.065 | 0.133 | 1 |
| **9930111J21Rik2** | 0.00715834 | -0.380063905 | 0.053 | 0.117 | 1 |
| **Inpp5d** | 0.007227705 | -0.441168273 | 0.078 | 0.148 | 1 |
| **Tut4** | 0.007229347 | -0.35803781 | 0.041 | 0.1 | 1 |
| **Il16** | 0.007553609 | 0.347066496 | 0.322 | 0.233 | 1 |
| **Ube2n** | 0.007722881 | 0.37499257 | 0.18 | 0.109 | 1 |
| **Skp1a** | 0.007833304 | -0.514073292 | 0.216 | 0.303 | 1 |
| **Etv6** | 0.007867584 | 0.348723224 | 0.11 | 0.053 | 1 |
| **Nsg2** | 0.007930232 | -0.3606045 | 0.073 | 0.143 | 1 |
| **Mrpl34** | 0.007939575 | 0.466658734 | 0.196 | 0.124 | 1 |
| **Rgcc** | 0.008006321 | -0.283755586 | 0.09 | 0.163 | 1 |
| **Bax** | 0.008117248 | 0.325367828 | 0.331 | 0.238 | 1 |
| **Selenok** | 0.00818875 | 0.36109049 | 0.441 | 0.357 | 1 |
| **Tasp1** | 0.008408142 | 0.399312164 | 0.106 | 0.051 | 1 |
| **Rwdd1** | 0.00864603 | 0.331944977 | 0.327 | 0.24 | 1 |
| **Snrpb** | 0.008773122 | 0.295444739 | 0.424 | 0.311 | 1 |
| **Pear1** | 0.008843647 | 0.298697666 | 0.11 | 0.053 | 1 |
| **Tmem176a** | 0.009236849 | -0.857922883 | 0.073 | 0.136 | 1 |
| **Tmem30a** | 0.00923702 | 0.391804834 | 0.163 | 0.095 | 1 |
| **Serpinb6a** | 0.009237913 | 0.347730199 | 0.188 | 0.114 | 1 |
| **Nav2** | 0.009503969 | 0.307203108 | 0.163 | 0.095 | 1 |
| **Arl5c** | 0.009571453 | -0.630954068 | 0.045 | 0.1 | 1 |
| **Trim8** | 0.009585539 | 0.354176215 | 0.176 | 0.104 | 1 |
| **Ergic3** | 0.009935125 | 0.357218994 | 0.22 | 0.143 | 1 |
| **Hspa8** | 0.009951626 | 0.307889673 | 0.784 | 0.762 | 1 |
| **Prdx2** | 0.010046704 | 0.410004965 | 0.331 | 0.243 | 1 |
| **Psma3** | 0.010096691 | 0.386278519 | 0.343 | 0.257 | 1 |
| **Psme1** | 0.010113328 | 0.301764285 | 0.608 | 0.49 | 1 |
| **Samhd1** | 0.010388416 | -0.407346614 | 0.253 | 0.342 | 1 |
| **Gnas** | 0.010424266 | 0.380173291 | 0.42 | 0.328 | 1 |
| **Ppp1r12a** | 0.010483701 | -0.543328504 | 0.143 | 0.214 | 1 |
| **Akirin1** | 0.010507687 | -0.451012673 | 0.057 | 0.117 | 1 |
| **Nt5e** | 0.01087764 | 0.345403362 | 0.155 | 0.09 | 1 |
| **Bag1** | 0.010922452 | 0.364901767 | 0.127 | 0.068 | 1 |
| **Lbh** | 0.010991061 | 0.353116026 | 0.408 | 0.318 | 1 |
| **Dnajc8** | 0.011098679 | 0.314400748 | 0.212 | 0.136 | 1 |
| **Ankrd12** | 0.011139449 | 0.351107723 | 0.265 | 0.182 | 1 |
| **Cnp** | 0.011327611 | -0.301096805 | 0.098 | 0.172 | 1 |
| **Nenf** | 0.011341531 | 0.365658428 | 0.151 | 0.087 | 1 |
| **Elavl1** | 0.011592042 | 0.364337 | 0.192 | 0.119 | 1 |
| **Eif5a** | 0.011648887 | 0.372584803 | 0.6 | 0.517 | 1 |
| **Stk38** | 0.011892488 | -0.480997076 | 0.094 | 0.16 | 1 |
| **Sptssa** | 0.012021762 | -0.538838836 | 0.196 | 0.277 | 1 |
| **Sec22b** | 0.012063876 | 0.339424639 | 0.106 | 0.053 | 1 |
| **Nfkbie** | 0.012189291 | 0.403344497 | 0.118 | 0.063 | 1 |
| **Rnaset2b** | 0.012231549 | 0.470021092 | 0.212 | 0.141 | 1 |
| **Rexo2** | 0.012680567 | -0.466980773 | 0.171 | 0.25 | 1 |
| **Macf1** | 0.012869716 | -0.438044904 | 0.143 | 0.216 | 1 |
| **Nfkbib** | 0.012994501 | 0.380224785 | 0.143 | 0.083 | 1 |
| **Rce1** | 0.013154679 | 0.327983326 | 0.102 | 0.051 | 1 |
| **H2afv** | 0.01330509 | 0.401597831 | 0.437 | 0.342 | 1 |
| **Atf1** | 0.013345932 | 0.33996136 | 0.135 | 0.075 | 1 |
| **Ppib** | 0.013762339 | 0.384964406 | 0.535 | 0.456 | 1 |
| **Txn2** | 0.013818316 | 0.359081158 | 0.302 | 0.223 | 1 |
| **Vps4b** | 0.013942753 | 0.381689473 | 0.151 | 0.09 | 1 |
| **Gm21988** | 0.013955194 | 0.289135562 | 0.102 | 0.051 | 1 |
| **Helz2** | 0.014011697 | 0.300836959 | 0.122 | 0.066 | 1 |
| **Trnau1ap** | 0.014036618 | 0.300027625 | 0.114 | 0.061 | 1 |
| **Sec11c** | 0.014042278 | 0.281675195 | 0.4 | 0.313 | 1 |
| **Rhof** | 0.014129307 | 0.259159074 | 0.22 | 0.143 | 1 |
| **Cited2** | 0.014180362 | 0.413646643 | 0.159 | 0.097 | 1 |
| **Zbtb1** | 0.01431675 | 0.446102933 | 0.118 | 0.066 | 1 |
| **Stambpl1** | 0.014838018 | -0.466451678 | 0.069 | 0.129 | 1 |
| **Wipf1** | 0.014856024 | 0.339466203 | 0.249 | 0.17 | 1 |
| **Bin1** | 0.015072954 | -0.416404388 | 0.057 | 0.114 | 1 |
| **Ubl4a** | 0.015137581 | 0.4162159 | 0.106 | 0.056 | 1 |
| **Izumo4** | 0.015160215 | 0.351189033 | 0.122 | 0.068 | 1 |
| **Sub1** | 0.015508026 | -0.366264974 | 0.38 | 0.454 | 1 |
| **Prkcb** | 0.015540757 | 0.334579323 | 0.127 | 0.07 | 1 |
| **Maea** | 0.016056996 | 0.308132317 | 0.127 | 0.07 | 1 |
| **Tsc22d3** | 0.016190024 | -0.455236438 | 0.143 | 0.218 | 1 |
| **Mrpl15** | 0.016197358 | 0.283622236 | 0.143 | 0.083 | 1 |
| **Snx2** | 0.016263049 | 0.294062899 | 0.163 | 0.1 | 1 |
| **Ppp1ca** | 0.016321782 | 0.312859053 | 0.498 | 0.42 | 1 |
| **Kmt2c** | 0.016358985 | -0.460036541 | 0.053 | 0.107 | 1 |
| **Fuca1** | 0.016409607 | 0.384162846 | 0.106 | 0.056 | 1 |
| **Ltb** | 0.016582776 | 0.293320822 | 0.78 | 0.733 | 1 |
| **Eef1akmt1** | 0.016789368 | 0.379845493 | 0.118 | 0.066 | 1 |
| **Arpc1b** | 0.016957943 | 0.252685819 | 0.665 | 0.578 | 1 |
| **Nde1** | 0.017195008 | 0.3104001 | 0.118 | 0.066 | 1 |
| **Paxx** | 0.017321195 | -0.532534448 | 0.049 | 0.1 | 1 |
| **Mfng** | 0.017404365 | 0.360069022 | 0.135 | 0.078 | 1 |
| **Cfap20** | 0.017551509 | 0.389950582 | 0.106 | 0.056 | 1 |
| **Rab5c** | 0.017621591 | 0.387444406 | 0.216 | 0.146 | 1 |
| **Evl** | 0.018158005 | -0.404099735 | 0.114 | 0.182 | 1 |
| **Usp16** | 0.018320197 | 0.276713752 | 0.106 | 0.056 | 1 |
| **Dctn6** | 0.018418352 | -0.365336868 | 0.082 | 0.143 | 1 |
| **Ly6a** | 0.018536416 | 0.343778142 | 0.596 | 0.517 | 1 |
| **Cbx6** | 0.018541909 | 0.388589492 | 0.114 | 0.063 | 1 |
| **Arid4a** | 0.018583742 | 0.521085088 | 0.188 | 0.121 | 1 |
| **Ptpn22** | 0.018623832 | 0.289583306 | 0.249 | 0.172 | 1 |
| **Zfp36l2** | 0.019568555 | -0.329233092 | 0.327 | 0.405 | 1 |
| **Rgs1** | 0.019678903 | -0.797755722 | 0.2 | 0.262 | 1 |
| **Tmem123** | 0.019795915 | -0.343671723 | 0.102 | 0.17 | 1 |
| **Rsbn1** | 0.019894588 | 0.289313029 | 0.122 | 0.068 | 1 |
| **Eif3h** | 0.0198952 | -0.269669232 | 0.551 | 0.626 | 1 |
| **Samsn1** | 0.019945853 | 0.255119261 | 0.233 | 0.155 | 1 |
| **Dars** | 0.019987134 | 0.359100033 | 0.159 | 0.1 | 1 |
| **Rpl13a** | 0.020073374 | -0.470081279 | 0.408 | 0.459 | 1 |
| **Yipf4** | 0.020100508 | 0.323350568 | 0.139 | 0.083 | 1 |
| **Naa38** | 0.020237936 | 0.382824387 | 0.22 | 0.153 | 1 |
| **Tcp1** | 0.020291609 | 0.388929328 | 0.216 | 0.15 | 1 |
| **1700123O20Rik** | 0.020590357 | 0.339596226 | 0.147 | 0.09 | 1 |
| **Ptges3** | 0.020701372 | 0.348310963 | 0.22 | 0.15 | 1 |
| **Emd** | 0.020717155 | 0.381917801 | 0.167 | 0.107 | 1 |
| **Arhgap9** | 0.021905703 | -0.310981645 | 0.114 | 0.184 | 1 |
| **Polr2j** | 0.022030813 | 0.373416751 | 0.196 | 0.133 | 1 |
| **Ankra2** | 0.022035727 | 0.376941967 | 0.131 | 0.078 | 1 |
| **Emp3** | 0.022099373 | -0.385633678 | 0.233 | 0.308 | 1 |
| **Fkbp4** | 0.022257853 | 0.460915848 | 0.171 | 0.114 | 1 |
| **Ikzf3** | 0.022269962 | -0.467201481 | 0.086 | 0.146 | 1 |
| **Tmem176b** | 0.022573001 | -0.835741408 | 0.09 | 0.146 | 1 |
| **Spr** | 0.022721506 | 0.276162392 | 0.171 | 0.109 | 1 |
| **Bloc1s1** | 0.022899142 | 0.279818126 | 0.273 | 0.199 | 1 |
| **Pacsin1** | 0.023001477 | 0.325643136 | 0.139 | 0.083 | 1 |
| **Mdp1** | 0.023012981 | -0.280596474 | 0.106 | 0.172 | 1 |
| **Ttc3** | 0.023212242 | 0.32420634 | 0.131 | 0.078 | 1 |
| **Cisd2** | 0.023361488 | 0.286754958 | 0.18 | 0.117 | 1 |
| **Uqcrc2** | 0.023587388 | 0.457791789 | 0.192 | 0.133 | 1 |
| **Smap2** | 0.023809893 | -0.334352118 | 0.057 | 0.109 | 1 |
| **Med8** | 0.024514513 | 0.309877738 | 0.135 | 0.08 | 1 |
| **4930523C07Rik** | 0.024600267 | -0.450097991 | 0.11 | 0.172 | 1 |
| **Stk10** | 0.024911946 | -0.430588786 | 0.127 | 0.194 | 1 |
| **Top2b** | 0.025115515 | 0.256596418 | 0.196 | 0.129 | 1 |
| **1810058I24Rik** | 0.02536085 | -0.37287512 | 0.233 | 0.311 | 1 |
| **Mtch2** | 0.025406321 | 0.3276608 | 0.147 | 0.09 | 1 |
| **Rapgef6** | 0.025918607 | -0.327185039 | 0.192 | 0.265 | 1 |
| **Tmbim6** | 0.026479228 | 0.392331029 | 0.461 | 0.386 | 1 |
| **Mbp** | 0.026563423 | -0.458521367 | 0.065 | 0.117 | 1 |
| **Tubb4b** | 0.026592775 | 0.40355803 | 0.188 | 0.126 | 1 |
| **Cbx7** | 0.027166838 | 0.275649914 | 0.155 | 0.097 | 1 |
| **Chd3** | 0.027536165 | -0.418374726 | 0.196 | 0.269 | 1 |
| **S100a6** | 0.027577941 | -0.728816633 | 0.355 | 0.42 | 1 |
| **Leprotl1** | 0.02766093 | -0.330613707 | 0.318 | 0.398 | 1 |
| **Cd3g** | 0.027727374 | 0.266264096 | 0.78 | 0.733 | 1 |
| **Msi2** | 0.027855936 | 0.345197223 | 0.147 | 0.092 | 1 |
| **Cd96** | 0.028093346 | -0.406683031 | 0.065 | 0.117 | 1 |
| **Snhg12** | 0.028108456 | -0.452484902 | 0.057 | 0.104 | 1 |
| **Ikbke** | 0.02837961 | -0.259734774 | 0.061 | 0.114 | 1 |
| **1-Sep** | 0.028543262 | -0.341942119 | 0.384 | 0.461 | 1 |
| **Xbp1** | 0.028602471 | 0.376560169 | 0.155 | 0.1 | 1 |
| **Ilk** | 0.029281976 | 0.352068828 | 0.159 | 0.102 | 1 |
| **Galnt6** | 0.029500272 | -0.315733184 | 0.057 | 0.107 | 1 |
| **Mrps23** | 0.029686011 | -0.355036362 | 0.118 | 0.182 | 1 |
| **Mrpl52** | 0.029943582 | 0.267785136 | 0.555 | 0.49 | 1 |
| **Atp6v1d** | 0.030163253 | 0.342129894 | 0.253 | 0.187 | 1 |
| **Fibp** | 0.03031588 | 0.346568124 | 0.118 | 0.07 | 1 |
| **Sirt2** | 0.030338997 | 0.356373196 | 0.163 | 0.107 | 1 |
| **Naa20** | 0.030923453 | 0.337338878 | 0.127 | 0.075 | 1 |
| **2410006H16Rik** | 0.031134076 | -0.392934979 | 0.384 | 0.464 | 1 |
| **Selenof** | 0.031253079 | 0.270373081 | 0.355 | 0.279 | 1 |
| **Smc6** | 0.031424702 | -0.376414984 | 0.094 | 0.15 | 1 |
| **Tmem106b** | 0.031661131 | 0.374204013 | 0.118 | 0.07 | 1 |
| **Ostf1** | 0.031881576 | -0.327861291 | 0.127 | 0.192 | 1 |
| **Atox1** | 0.031968975 | 0.408149381 | 0.38 | 0.316 | 1 |
| **Vsir** | 0.031994728 | 0.273726494 | 0.171 | 0.112 | 1 |
| **Gm9794** | 0.032270852 | -0.338091484 | 0.065 | 0.117 | 1 |
| **Ddx50** | 0.032903432 | -0.342771033 | 0.09 | 0.146 | 1 |
| **Pias1** | 0.033061829 | -0.361929276 | 0.061 | 0.112 | 1 |
| **St13** | 0.033377502 | 0.312377896 | 0.257 | 0.187 | 1 |
| **Rab8b** | 0.033558075 | -0.303624371 | 0.065 | 0.117 | 1 |
| **Elk3** | 0.033798547 | 0.393325667 | 0.122 | 0.075 | 1 |
| **Ssrp1** | 0.033977883 | 0.351484004 | 0.171 | 0.117 | 1 |
| **Cmtm7** | 0.034003432 | -0.378192199 | 0.143 | 0.206 | 1 |
| **Mau2** | 0.034029571 | 0.331138183 | 0.102 | 0.058 | 1 |
| **Irf7** | 0.034479857 | 0.314403274 | 0.188 | 0.129 | 1 |
| **Prelid3b** | 0.034526431 | 0.307641799 | 0.176 | 0.117 | 1 |
| **Rpl7l1** | 0.034950725 | 0.384548782 | 0.118 | 0.07 | 1 |
| **Sri** | 0.035100357 | 0.263470827 | 0.384 | 0.311 | 1 |
| **Arl6ip1** | 0.035569131 | -0.299824461 | 0.371 | 0.444 | 1 |
| **Slc25a39** | 0.036436771 | 0.403879655 | 0.192 | 0.136 | 1 |
| **Fermt3** | 0.036484121 | 0.322383213 | 0.257 | 0.189 | 1 |
| **Rbm8a** | 0.036509349 | 0.273742437 | 0.184 | 0.124 | 1 |
| **Wdr1** | 0.036573305 | 0.420495982 | 0.265 | 0.204 | 1 |
| **Pdcd6ip** | 0.036877251 | 0.382711562 | 0.155 | 0.102 | 1 |
| **Tm2d1** | 0.037092646 | 0.256170762 | 0.118 | 0.07 | 1 |
| **Gimap6** | 0.037279137 | -0.40207869 | 0.318 | 0.391 | 1 |
| **Atp6v0b** | 0.037679949 | 0.369235493 | 0.196 | 0.138 | 1 |
| **Sumo2** | 0.037855022 | 0.317946677 | 0.543 | 0.478 | 1 |
| **Ebna1bp2** | 0.038360565 | 0.273476482 | 0.151 | 0.097 | 1 |
| **Anp32a** | 0.038472508 | -0.406445645 | 0.31 | 0.371 | 1 |
| **Zbtb8os** | 0.038529861 | 0.316169834 | 0.114 | 0.068 | 1 |
| **Aes** | 0.038714213 | -0.392140936 | 0.22 | 0.286 | 1 |
| **Etfa** | 0.039012385 | 0.299894068 | 0.139 | 0.087 | 1 |
| **Lsp1** | 0.039203055 | -0.400098671 | 0.351 | 0.41 | 1 |
| **Selenos** | 0.039253543 | 0.446169752 | 0.237 | 0.177 | 1 |
| **Cyth1** | 0.041166818 | -0.466841168 | 0.09 | 0.141 | 1 |
| **Smarce1** | 0.041486969 | 0.401482052 | 0.122 | 0.078 | 1 |
| **Phb2** | 0.041953598 | 0.279660012 | 0.257 | 0.194 | 1 |
| **Dguok** | 0.042028281 | 0.283920753 | 0.155 | 0.102 | 1 |
| **Camk2d** | 0.042105948 | 0.302408034 | 0.176 | 0.117 | 1 |
| **Rap1a** | 0.04328773 | 0.345858618 | 0.331 | 0.267 | 1 |
| **Pstpip1** | 0.043377753 | 0.313746518 | 0.159 | 0.107 | 1 |
| **Itm2b** | 0.043685943 | 0.38454448 | 0.796 | 0.752 | 1 |
| **Calr** | 0.044786742 | 0.267044707 | 0.282 | 0.211 | 1 |
| **Cab39** | 0.045176917 | 0.308337279 | 0.143 | 0.092 | 1 |
| **Zcchc7** | 0.0454196 | 0.345066675 | 0.106 | 0.063 | 1 |
| **Kdm5b** | 0.045491827 | -0.368202996 | 0.065 | 0.112 | 1 |
| **Gm10177** | 0.046064037 | 0.274882702 | 0.127 | 0.08 | 1 |
| **Tmbim4** | 0.04616066 | 0.350842091 | 0.204 | 0.146 | 1 |
| **Gpx1** | 0.046455116 | -0.352876771 | 0.4 | 0.468 | 1 |
| **Tapbp** | 0.046464663 | -0.369773475 | 0.171 | 0.235 | 1 |
| **Fam189b** | 0.046672432 | -0.283829157 | 0.151 | 0.211 | 1 |
| **Eif1b** | 0.046733925 | 0.352142465 | 0.184 | 0.129 | 1 |
| **Stap1** | 0.046842101 | 0.359179896 | 0.102 | 0.061 | 1 |
| **Pdap1** | 0.04691639 | 0.259711107 | 0.176 | 0.121 | 1 |
| **Rtf1** | 0.047003186 | -0.421112374 | 0.061 | 0.104 | 1 |
| **Pld3** | 0.047140438 | 0.296425368 | 0.155 | 0.104 | 1 |
| **Cdc123** | 0.047818429 | 0.322399344 | 0.114 | 0.07 | 1 |
| **Lims1** | 0.049226468 | 0.270446867 | 0.131 | 0.083 | 1 |
| **Trbv13-3** | 0.050585548 | 0.779412549 | 0.106 | 0.066 | 1 |
| **Rnf166** | 0.050728614 | -0.287562368 | 0.061 | 0.107 | 1 |
| **Spata13** | 0.050848615 | 0.286394883 | 0.167 | 0.114 | 1 |
| **Babam1** | 0.051020061 | 0.277955353 | 0.139 | 0.09 | 1 |
| **Birc2** | 0.051070053 | 0.267143072 | 0.147 | 0.097 | 1 |
| **Nmi** | 0.05132972 | 0.258101044 | 0.135 | 0.087 | 1 |
| **Ikzf1** | 0.051561699 | -0.39776393 | 0.135 | 0.192 | 1 |
| **Hpcal1** | 0.051721448 | 0.442688515 | 0.188 | 0.136 | 1 |
| **Twf2** | 0.052256899 | -0.273132105 | 0.098 | 0.15 | 1 |
| **Crlf2** | 0.052459637 | 0.486689311 | 0.2 | 0.148 | 1 |
| **Ctsd** | 0.052540592 | -0.359368663 | 0.094 | 0.146 | 1 |
| **Rest** | 0.053106392 | 0.315639738 | 0.11 | 0.068 | 1 |
| **Fryl** | 0.053561271 | 0.271162843 | 0.2 | 0.143 | 1 |
| **Tgfbr2** | 0.054041066 | -0.332084082 | 0.098 | 0.15 | 1 |
| **Mrps21** | 0.055257495 | 0.268544804 | 0.376 | 0.308 | 1 |
| **Nr4a1** | 0.055545332 | 0.513466944 | 0.11 | 0.068 | 1 |
| **Prkcsh** | 0.055701302 | 0.296652023 | 0.131 | 0.085 | 1 |
| **Vmp1** | 0.056221754 | 0.292869003 | 0.171 | 0.119 | 1 |
| **Sap18** | 0.056826206 | 0.267886518 | 0.363 | 0.296 | 1 |
| **Ankrd11** | 0.057224546 | -0.334575775 | 0.122 | 0.177 | 1 |
| **Lcp2** | 0.057256383 | 0.309591146 | 0.327 | 0.265 | 1 |
| **Ech1** | 0.057482107 | 0.27079593 | 0.184 | 0.131 | 1 |
| **Pfkp** | 0.057683784 | 0.347618457 | 0.127 | 0.083 | 1 |
| **Zfp706** | 0.058461711 | -0.250237145 | 0.278 | 0.352 | 1 |
| **Psmb10** | 0.059152304 | 0.263744594 | 0.261 | 0.199 | 1 |
| **Papola** | 0.059189747 | 0.261598379 | 0.188 | 0.133 | 1 |
| **Itga4** | 0.059574546 | -0.419139744 | 0.131 | 0.182 | 1 |
| **Higd1a** | 0.060335505 | 0.4025184 | 0.233 | 0.177 | 1 |
| **Ik** | 0.06033646 | -0.345304148 | 0.078 | 0.124 | 1 |
| **Tspan32** | 0.060711736 | 0.251054246 | 0.396 | 0.335 | 1 |
| **Gtf2i** | 0.060893016 | 0.345395181 | 0.167 | 0.119 | 1 |
| **Faap20** | 0.060910529 | 0.273248994 | 0.155 | 0.107 | 1 |
| **Vdac1** | 0.061013362 | 0.259978063 | 0.2 | 0.143 | 1 |
| **Tmem147** | 0.061058511 | 0.274627865 | 0.106 | 0.066 | 1 |
| **Adcy7** | 0.061340354 | -0.367934393 | 0.098 | 0.148 | 1 |
| **Ubr4** | 0.062254617 | 0.261207068 | 0.114 | 0.073 | 1 |
| **Cdc42ep3** | 0.062668863 | -0.360268571 | 0.065 | 0.107 | 1 |
| **Mrfap1** | 0.062669996 | 0.311478509 | 0.351 | 0.282 | 1 |
| **Vps36** | 0.06293605 | 0.289653551 | 0.122 | 0.08 | 1 |
| **Ip6k2** | 0.063201668 | 0.254702462 | 0.106 | 0.066 | 1 |
| **Casp8** | 0.063389347 | 0.348201011 | 0.143 | 0.097 | 1 |
| **Glrx3** | 0.063505457 | 0.25849992 | 0.155 | 0.107 | 1 |
| **Uqcr10** | 0.063875379 | 0.268544646 | 0.461 | 0.403 | 1 |
| **Myo1g** | 0.064006542 | -0.328945834 | 0.078 | 0.124 | 1 |
| **Ccm2** | 0.064457984 | 0.324226842 | 0.188 | 0.136 | 1 |
| **N4bp2l2** | 0.065107158 | -0.388545994 | 0.065 | 0.107 | 1 |
| **Sdcbp** | 0.065330322 | 0.279827543 | 0.184 | 0.133 | 1 |
| **Aip** | 0.065475674 | 0.274095478 | 0.18 | 0.129 | 1 |
| **Taf11** | 0.065883399 | 0.390190273 | 0.11 | 0.07 | 1 |
| **Rtraf** | 0.066514051 | 0.280043594 | 0.408 | 0.354 | 1 |
| **Apobec3** | 0.066556584 | 0.262593343 | 0.38 | 0.308 | 1 |
| **Elf2** | 0.066684222 | 0.250927408 | 0.155 | 0.107 | 1 |
| **Cops6** | 0.06668664 | 0.27128225 | 0.2 | 0.148 | 1 |
| **Smndc1** | 0.066747619 | 0.318808904 | 0.212 | 0.158 | 1 |
| **Sec11a** | 0.066945468 | -0.311498038 | 0.151 | 0.201 | 1 |
| **Ppp2r5c** | 0.067557336 | 0.37461703 | 0.176 | 0.129 | 1 |
| **Ten1** | 0.068258162 | -0.395884852 | 0.078 | 0.121 | 1 |
| **Immp1l** | 0.068994411 | 0.285210939 | 0.122 | 0.08 | 1 |
| **Def6** | 0.070013811 | -0.405542727 | 0.139 | 0.189 | 1 |
| **Nsa2** | 0.070223279 | -0.27927871 | 0.392 | 0.454 | 1 |
| **Spag7** | 0.070251502 | 0.293894752 | 0.131 | 0.087 | 1 |
| **Sf3b6** | 0.07041499 | 0.311460344 | 0.351 | 0.291 | 1 |
| **Pink1** | 0.070467009 | 0.256257995 | 0.102 | 0.063 | 1 |
| **Cct6a** | 0.071970479 | -0.432047166 | 0.102 | 0.15 | 1 |
| **Klf3** | 0.073091908 | -0.465393265 | 0.073 | 0.114 | 1 |
| **Ak2** | 0.073284355 | 0.267667048 | 0.196 | 0.141 | 1 |
| **Tmc6** | 0.07336572 | 0.32117865 | 0.102 | 0.066 | 1 |
| **Nsmce1** | 0.073656324 | -0.326884808 | 0.061 | 0.102 | 1 |
| **Spop** | 0.075238799 | 0.285395544 | 0.188 | 0.138 | 1 |
| **Rpn2** | 0.077667543 | 0.286363983 | 0.135 | 0.092 | 1 |
| **Lsm3** | 0.077706638 | 0.258068661 | 0.176 | 0.126 | 1 |
| **Ndufa12** | 0.079044923 | 0.352371185 | 0.147 | 0.104 | 1 |
| **Polr2f** | 0.079172349 | 0.404844802 | 0.192 | 0.148 | 1 |
| **Nucb1** | 0.07931463 | 0.311111542 | 0.151 | 0.107 | 1 |
| **Smarca5** | 0.079567831 | 0.300561403 | 0.131 | 0.087 | 1 |
| **Trbv19** | 0.079787493 | 0.571003067 | 0.114 | 0.075 | 1 |
| **Sin3b** | 0.080459604 | 0.261524203 | 0.204 | 0.153 | 1 |
| **Dnajc1** | 0.081004477 | 0.354706834 | 0.122 | 0.083 | 1 |
| **Sirt7** | 0.081454157 | -0.318286974 | 0.11 | 0.16 | 1 |
| **Arf1** | 0.082816174 | 0.255905273 | 0.408 | 0.342 | 1 |
| **Plekhb2** | 0.082845925 | 0.392391893 | 0.118 | 0.08 | 1 |
| **Ndufa1** | 0.083394956 | 0.269545688 | 0.376 | 0.316 | 1 |
| **4932438A13Rik** | 0.083419365 | -0.346231 | 0.086 | 0.129 | 1 |
| **Tomm22** | 0.084393302 | -0.264463567 | 0.265 | 0.328 | 1 |
| **Atxn2l** | 0.085269692 | -0.457902401 | 0.098 | 0.141 | 1 |
| **H1f0** | 0.085394717 | 0.256484771 | 0.114 | 0.075 | 1 |
| **Ramp1** | 0.085745668 | -0.317987057 | 0.278 | 0.335 | 1 |
| **Rgs19** | 0.086446639 | -0.288172784 | 0.094 | 0.138 | 1 |
| **Tmem179b** | 0.08667888 | 0.282612585 | 0.302 | 0.25 | 1 |
| **Mycbp2** | 0.0872518 | -0.370302011 | 0.139 | 0.187 | 1 |
| **Sp140** | 0.087645716 | 0.328886767 | 0.139 | 0.097 | 1 |
| **Tmed5** | 0.090379884 | -0.286589109 | 0.082 | 0.124 | 1 |
| **Tuba1b** | 0.091045242 | 0.332790075 | 0.233 | 0.18 | 1 |
| **Herpud1** | 0.091125807 | 0.329186732 | 0.11 | 0.073 | 1 |
| **Irf2bpl** | 0.091480799 | -0.361290543 | 0.065 | 0.104 | 1 |
| **Cd69** | 0.092449361 | 0.275623992 | 0.188 | 0.138 | 1 |
| **Elmo1** | 0.093089605 | 0.272334223 | 0.102 | 0.066 | 1 |
| **Ncor1** | 0.0935171 | -0.268793256 | 0.204 | 0.265 | 1 |
| **Mtf2** | 0.093573392 | -0.288110539 | 0.061 | 0.1 | 1 |
| **Mrpl45** | 0.094067235 | 0.329398116 | 0.106 | 0.07 | 1 |
| **Kxd1** | 0.094717861 | 0.336823626 | 0.224 | 0.177 | 1 |
| **Glrx** | 0.094815663 | -0.323340748 | 0.114 | 0.16 | 1 |
| **Acp1** | 0.095371395 | 0.26035128 | 0.127 | 0.087 | 1 |
| **Lgals9** | 0.095432752 | -0.374503781 | 0.167 | 0.214 | 1 |
| **Ran** | 0.095676125 | 0.315218127 | 0.408 | 0.357 | 1 |
| **Rbm38** | 0.098000058 | -0.277993376 | 0.212 | 0.262 | 1 |
| **Tbcb** | 0.099252013 | 0.347483305 | 0.18 | 0.136 | 1 |
| **Nfkb1** | 0.100803444 | 0.252212319 | 0.188 | 0.138 | 1 |
| **Bptf** | 0.100859798 | -0.294668375 | 0.11 | 0.155 | 1 |
| **Rsrp1** | 0.104365105 | -0.25406169 | 0.331 | 0.379 | 1 |
| **Uimc1** | 0.10575348 | 0.380366924 | 0.139 | 0.102 | 1 |
| **Cdkn1b** | 0.108450905 | -0.362039562 | 0.106 | 0.148 | 1 |
| **Tex264** | 0.108584439 | -0.28900668 | 0.078 | 0.117 | 1 |
| **Tpst2** | 0.108601609 | -0.26633402 | 0.114 | 0.158 | 1 |
| **Fam169b** | 0.10880562 | 0.261195909 | 0.151 | 0.107 | 1 |
| **Il6st** | 0.108885041 | 0.253089666 | 0.131 | 0.09 | 1 |
| **Rpl31** | 0.110981149 | -0.25469885 | 0.49 | 0.522 | 1 |
| **Ube2e1** | 0.114381443 | 0.293277924 | 0.143 | 0.104 | 1 |
| **Ldb1** | 0.116136189 | -0.306370642 | 0.114 | 0.158 | 1 |
| **Fkbp2** | 0.116273045 | 0.406338084 | 0.122 | 0.087 | 1 |
| **Smarcb1** | 0.117441532 | 0.295214013 | 0.131 | 0.092 | 1 |
| **Sla2** | 0.118420735 | 0.307938923 | 0.127 | 0.09 | 1 |
| **Pop7** | 0.118734332 | 0.285656797 | 0.106 | 0.073 | 1 |
| **Mettl23** | 0.119020621 | 0.328637942 | 0.314 | 0.267 | 1 |
| **Ddx17** | 0.119509973 | -0.360829184 | 0.102 | 0.141 | 1 |
| **Vcp** | 0.119743183 | 0.261843848 | 0.278 | 0.223 | 1 |
| **Cbl** | 0.119935637 | -0.332154835 | 0.106 | 0.148 | 1 |
| **Eif1ad** | 0.120275771 | -0.344779794 | 0.069 | 0.104 | 1 |
| **Hdlbp** | 0.12091351 | 0.278708549 | 0.118 | 0.083 | 1 |
| **Cox14** | 0.130309571 | 0.374484724 | 0.261 | 0.221 | 1 |
| **Zcchc9** | 0.130516633 | 0.272773011 | 0.114 | 0.08 | 1 |
| **Fam53b** | 0.130742374 | -0.323266924 | 0.09 | 0.129 | 1 |
| **Fam173a** | 0.131210717 | -0.31959894 | 0.155 | 0.201 | 1 |
| **Bcas2** | 0.135574217 | 0.259316571 | 0.204 | 0.16 | 1 |
| **Vps29** | 0.138480316 | 0.338528564 | 0.131 | 0.097 | 1 |
| **Ier5** | 0.140262627 | 0.299211888 | 0.184 | 0.143 | 1 |
| **Plec** | 0.140339107 | 0.251603403 | 0.159 | 0.119 | 1 |
| **Dnaja2** | 0.140625499 | 0.359869158 | 0.176 | 0.136 | 1 |
| **Rchy1** | 0.140967144 | -0.372236681 | 0.098 | 0.133 | 1 |
| **Trp53inp1** | 0.14142639 | 0.383457168 | 0.131 | 0.097 | 1 |
| **Cct8** | 0.142144979 | 0.367452948 | 0.163 | 0.126 | 1 |
| **Rer1** | 0.142337671 | 0.292355929 | 0.216 | 0.177 | 1 |
| **Dnajc19** | 0.143016743 | -0.277900782 | 0.131 | 0.172 | 1 |
| **Ndrg3** | 0.144653643 | -0.301475987 | 0.073 | 0.107 | 1 |
| **Prpf38b** | 0.145559594 | 0.260705168 | 0.163 | 0.121 | 1 |
| **Ddb1** | 0.146946413 | 0.30212075 | 0.122 | 0.09 | 1 |
| **Zfp148** | 0.148797618 | -0.331964221 | 0.069 | 0.102 | 1 |
| **Arl1** | 0.149280519 | 0.29301827 | 0.11 | 0.078 | 1 |
| **Jak2** | 0.150250015 | 0.279734314 | 0.11 | 0.078 | 1 |
| **Tecpr1** | 0.151702278 | -0.339734011 | 0.159 | 0.201 | 1 |
| **Il2ra** | 0.153017084 | -0.251846386 | 0.098 | 0.136 | 1 |
| **Ephx1** | 0.15536716 | 0.304036295 | 0.155 | 0.119 | 1 |
| **Selenop** | 0.155515574 | -0.445107278 | 0.135 | 0.17 | 1 |
| **Mta2** | 0.155682149 | 0.260301479 | 0.155 | 0.119 | 1 |
| **Kdm5c** | 0.160855298 | 0.296117108 | 0.102 | 0.073 | 1 |
| **Emb** | 0.161266876 | -0.342236323 | 0.229 | 0.267 | 1 |
| **Gimap9** | 0.163891328 | 0.305797699 | 0.159 | 0.124 | 1 |
| **Zyx** | 0.163966863 | -0.258991169 | 0.127 | 0.165 | 1 |
| **Cope** | 0.164183769 | 0.274924374 | 0.208 | 0.17 | 1 |
| **Hsp90b1** | 0.165064607 | -0.295359817 | 0.249 | 0.294 | 1 |
| **1110038B12Rik** | 0.167037831 | -0.312888215 | 0.102 | 0.136 | 1 |
| **Smc4** | 0.167211569 | -0.475901037 | 0.298 | 0.32 | 1 |
| **Srpr** | 0.168652485 | 0.25651481 | 0.114 | 0.083 | 1 |
| **Safb2** | 0.170286961 | 0.384192785 | 0.122 | 0.092 | 1 |
| **Tap2** | 0.172114167 | -0.334681475 | 0.184 | 0.223 | 1 |
| **Ubash3a** | 0.176681682 | -0.325376616 | 0.098 | 0.131 | 1 |
| **Cyb5a** | 0.181757311 | -0.306292766 | 0.257 | 0.308 | 1 |
| **Trappc6a** | 0.181975458 | 0.26054757 | 0.163 | 0.126 | 1 |
| **BC004004** | 0.182543832 | 0.262486305 | 0.147 | 0.112 | 1 |
| **Denr** | 0.184111103 | 0.291209487 | 0.171 | 0.136 | 1 |
| **Eif4e** | 0.18523541 | 0.282287595 | 0.11 | 0.08 | 1 |
| **Diaph1** | 0.190392427 | -0.251806292 | 0.09 | 0.121 | 1 |
| **Mllt3** | 0.19286681 | -0.266263878 | 0.102 | 0.136 | 1 |
| **Cetn2** | 0.19377304 | 0.284546024 | 0.131 | 0.1 | 1 |
| **Tspan13** | 0.198582406 | -0.29987664 | 0.273 | 0.308 | 1 |
| **Mier1** | 0.199253935 | -0.31184422 | 0.188 | 0.226 | 1 |
| **Ccdc88c** | 0.199359451 | -0.284204707 | 0.082 | 0.112 | 1 |
| **Snrpb2** | 0.201918218 | -0.296245379 | 0.09 | 0.121 | 1 |
| **Zcrb1** | 0.20602393 | 0.378991845 | 0.2 | 0.172 | 1 |
| **Sbno1** | 0.214041023 | 0.284152564 | 0.147 | 0.117 | 1 |
| **Romo1** | 0.215347469 | 0.294366437 | 0.249 | 0.214 | 1 |
| **Rbm6** | 0.216303529 | 0.262545106 | 0.11 | 0.083 | 1 |
| **Prex1** | 0.217802792 | -0.280186705 | 0.098 | 0.129 | 1 |
| **Snrnp27** | 0.219230487 | 0.351479874 | 0.143 | 0.114 | 1 |
| **Ubxn1** | 0.222764495 | -0.263128666 | 0.29 | 0.325 | 1 |
| **Arpc4** | 0.223436218 | 0.258301057 | 0.335 | 0.294 | 1 |
| **Tmem9b** | 0.225195045 | -0.264604978 | 0.118 | 0.15 | 1 |
| **Gbp4** | 0.225636139 | -0.269456589 | 0.073 | 0.102 | 1 |
| **Baz1b** | 0.225724969 | -0.290429036 | 0.078 | 0.104 | 1 |
| **Zfp36** | 0.226131523 | -0.292303471 | 0.318 | 0.35 | 1 |
| **Hspd1** | 0.226505339 | 0.266198111 | 0.18 | 0.148 | 1 |
| **Bmyc** | 0.229762845 | 0.464974933 | 0.224 | 0.192 | 1 |
| **Dpm3** | 0.230354847 | -0.340469068 | 0.204 | 0.238 | 1 |
| **Ncoa7** | 0.236778291 | -0.305284957 | 0.082 | 0.109 | 1 |
| **Rad21** | 0.242148289 | 0.251632864 | 0.147 | 0.117 | 1 |
| **Il27ra** | 0.246353568 | 0.262466661 | 0.196 | 0.165 | 1 |
| **Pttg1** | 0.249604822 | -0.305710581 | 0.114 | 0.143 | 1 |
| **Pum2** | 0.254685388 | -0.255793148 | 0.114 | 0.143 | 1 |
| **Srsf4** | 0.255042173 | -0.287082663 | 0.09 | 0.117 | 1 |
| **Fus** | 0.258556287 | -0.308078864 | 0.188 | 0.218 | 1 |
| **Glmp** | 0.261036567 | 0.327165262 | 0.18 | 0.153 | 1 |
| **Gpr68** | 0.27052512 | 0.251464981 | 0.114 | 0.09 | 1 |
| **Gm9493** | 0.271628278 | -0.266726514 | 0.098 | 0.124 | 1 |
| **Mrps16** | 0.272177641 | 0.27528903 | 0.282 | 0.255 | 1 |
| **Pak2** | 0.283445381 | -0.287347314 | 0.318 | 0.333 | 1 |
| **Slamf6** | 0.286362949 | -0.295877685 | 0.188 | 0.221 | 1 |
| **B4galnt1** | 0.289817994 | -0.25506298 | 0.269 | 0.299 | 1 |
| **Dock10** | 0.291928489 | -0.255848376 | 0.098 | 0.124 | 1 |
| **Cd27** | 0.292196918 | 0.301540919 | 0.376 | 0.347 | 1 |
| **Banf1** | 0.294360434 | -0.26598518 | 0.261 | 0.289 | 1 |
| **Nrip1** | 0.300173546 | -0.265087052 | 0.102 | 0.126 | 1 |
| **Lrmp** | 0.31938388 | 0.252167547 | 0.118 | 0.097 | 1 |
| **Btg2** | 0.328399391 | -0.286200152 | 0.404 | 0.427 | 1 |
| **Dctn2** | 0.329118228 | 0.303392446 | 0.122 | 0.102 | 1 |
| **Asap1** | 0.337533974 | -0.266687878 | 0.078 | 0.1 | 1 |
| **Cd5** | 0.337780743 | -0.289849417 | 0.245 | 0.272 | 1 |
| **Igbp1** | 0.344996126 | -0.257055373 | 0.09 | 0.112 | 1 |
| **Tmco1** | 0.365313183 | -0.263074201 | 0.09 | 0.109 | 1 |
| **Srpk1** | 0.371484041 | -0.294217901 | 0.176 | 0.199 | 1 |
| **Icos** | 0.377827971 | -0.263954976 | 0.249 | 0.277 | 1 |
| **Mat2a** | 0.395225497 | 0.346619926 | 0.147 | 0.126 | 1 |
| **Isg20** | 0.417646393 | 0.306568902 | 0.127 | 0.109 | 1 |
| **Atp2b1** | 0.476783591 | -0.255480509 | 0.163 | 0.182 | 1 |
| **Usf2** | 0.510260047 | 0.254191243 | 0.118 | 0.104 | 1 |
| **2310009A05Rik** | 0.533210563 | 0.269711983 | 0.102 | 0.09 | 1 |
| **Rsrc2** | 0.53635485 | -0.250709669 | 0.171 | 0.184 | 1 |
| **Mkrn1** | 0.539050988 | -0.267434084 | 0.122 | 0.136 | 1 |
| **Gpr183** | 0.548144495 | -0.289281318 | 0.114 | 0.126 | 1 |
| **Neat1** | 0.661788497 | 0.264669253 | 0.131 | 0.124 | 1 |
| **Gatad2b** | 0.729153637 | -0.250465472 | 0.139 | 0.146 | 1 |
| **Jun** | 0.963786865 | -0.31468098 | 0.127 | 0.121 | 1 |

Table S4. Fluorochrome-conjugated antibodies used in flow cytometry

| **Antibody** | **Supplier** | **Catalogue** | **RRID** |
| --- | --- | --- | --- |
| Anti-CD69 Antibody, FITC, Clone H1.2F3 | BioLegend | 104506 | AB_313109 |
| Anti-CD3 Antibody, PE-CF594, Clone 145-2C11 | BD Biosciences | 562097 | AB_11153307 |
| Anti-CD8 Antibody, BV421, Clone 53-6.7 | BD Biosciences | 563898 | AB_2738474 |
| Anti-CD4 Antibody, PE/Cyanine7, Clone GK1.5 | BioLegend | 100422 | AB_312707 |
| Anti-CD25 Antibody, APC/Cyanine7, Clone PC61 | BioLegend | 102026 | AB_830745 |
| Anti-FoxP3 Antibody, PE, Clone MF-14 | BioLegend | 126404 | AB_1089117 |
| Anti-H2-Kb Antibody,BV650, Clone AF6-88.5 | BD Biosciences | 742861 | AB_2741103 |
| Anti-IFN-γ Antibody, PE, Clone XMG1.2 | BioLegend | 505806 | AB_315400 |
| Anti-pSTAT1 Antibody, AF488, Clone A15158B | BioLegend | 686410 | AB_2650784 |
| Anti-IL-10 Antibody, APC, Clone JES5-16E3 | BioLegend | 505010 | AB_315364 |
| Anti- TNF-α Antibody, PE/Cyanine7, Clone MP6-XT22 | BioLegend | 506324 | AB_2256076 |
| Anti-IL-17A Antibody, APC/Cyanine7, Clone TC11-18H10.1 | BioLegend | 506940 | AB_2565781 |
| Anti-H2-Kd Antibody, FITC, Clone SF1-1.1 | BioLegend | 116606 | AB_313741 |
| Anti-CD45.2 Antibody, APC, Clone 104 | BioLegend | 109814 | AB_389211 |
| Anti-TIGIT Antibody, PE, Clone A17200C | BioLegend | 622206 | AB_2832838 |
| Anti-CD121b Antibody, PE, Clone 4E2 | BD Biosciences | 554450 | AB_395399 |
| Anti-CD45.1 Antibody, APC/Cyanine7, Clone A20 | BioLegend | 110716 | AB_313505 |
| Anti-TIGIT Antibody, AF647, Clone A15153G | BioLegend | 372724 | AB_2715972 |
| Anti-CD1c Antibody, FITC, Clone L161 | BioLegend | 331518 | AB_2073403 |
| Anti-CD14 Antibody, APC/Cyanine7, Clone 63D3 | BioLegend | 367108 | AB_2566710 |
| Anti-IL1R2 Antibody, FITC, Clone34141 | Invitrogen | MA5-23662 | AB_2609078 |
| Anti-CD3 Antibody, PE-CF594, Clone UCHT1 | BD Biosciences | 562280 | AB_11153674 |
| Anti-CD45RA Antibody, BV421, Clone 5H9 | BD Biosciences | 740083 | AB_2739846 |
| Anti- IL-27p28 Antibody, eFluor 660, Clone 3D1p28 | eBioscience | 50-8277-41 |  |
